# Fully Three-Dimensional Hemodynamic Characterization of Altered Blood Flow in Bicuspid Aortic Valve Patients With Respect to Aortic Dilatation: A Finite Element Approach

**DOI:** 10.3389/fcvm.2022.885338

**Published:** 2022-05-18

**Authors:** Julio Sotelo, Pamela Franco, Andrea Guala, Lydia Dux-Santoy, Aroa Ruiz-Muñoz, Arturo Evangelista, Hernan Mella, Joaquín Mura, Daniel E. Hurtado, José F. Rodríguez-Palomares, Sergio Uribe

**Affiliations:** ^1^School of Biomedical Engineering, Universidad de Valparaíso, Valparaíso, Chile; ^2^Biomedical Imaging Center, Pontificia Universidad Católica de Chile, Santiago, Chile; ^3^Millennium Institute for Intelligent Healthcare Engineering, iHEALTH, Santiago, Chile; ^4^Millennium Nucleus in Cardiovascular Magnetic Resonance, Cardio MR, Santiago, Chile; ^5^Department of Electrical Engineering, Pontificia Universidad Católica de Chile, Santiago, Chile; ^6^Department of Cardiology, Hospital Universitari Vall d'Hebron, CIBER-CV, Vall d'Hebron Institut de Recerca (VHIR), Barcelona, Spain; ^7^Department of Mechanical Engineering, Universidad Técnica Federico Santa María, Santiago, Chile; ^8^Department of Structural and Geotechnical Engineering, Pontificia Universidad Católica de Chile, Santiago, Chile; ^9^Institute for Biological and Medical Engineering, Schools of Engineering, Medicine and Biological Sciences, Pontificia Universidad Católica de Chile, Santiago, Chile; ^10^Department of Radiology, Schools of Medicine, Pontificia Universidad Católica de Chile, Santiago, Chile

**Keywords:** 4D flow CMR, finite elements, hemodynamics parameters, Bicuspid aortic valve, congenital heart disease, aneurysm, magnetic resonance imaging (MRI), vascular disease

## Abstract

**Background and Purpose:**

Prognostic models based on cardiovascular hemodynamic parameters may bring new information for an early assessment of patients with bicuspid aortic valve (BAV), playing a key role in reducing the long-term risk of cardiovascular events. This work quantifies several three-dimensional hemodynamic parameters in different patients with BAV and ranks their relationships with aortic diameter.

**Materials and Methods:**

Using 4D-flow CMR data of 74 patients with BAV (49 right-left and 25 right-non-coronary) and 48 healthy volunteers, aortic 3D maps of seventeen 17 different hemodynamic parameters were quantified along the thoracic aorta. Patients with BAV were divided into two morphotype categories, BAV-Non-AAoD (where we include 18 non-dilated patients and 7 root-dilated patients) and BAV-AAoD (where we include the 49 patients with dilatation of the ascending aorta). Differences between volunteers and patients were evaluated using MANOVA with Pillai's trace statistic, Mann–Whitney U test, ROC curves, and minimum redundancy maximum relevance algorithm. Spearman's correlation was used to correlate the dilation with each hemodynamic parameter.

**Results:**

The flow eccentricity, backward velocity, velocity angle, regurgitation fraction, circumferential wall shear stress, axial vorticity, and axial circulation allowed to discriminate between volunteers and patients with BAV, even in the absence of dilation. In patients with BAV, the diameter presented a strong correlation (> |+/−0.7|) with the forward velocity and velocity angle, and a good correlation (> |+/−0.5|) with regurgitation fraction, wall shear stress, wall shear stress axial, and vorticity, also for morphotypes and phenotypes, some of them are correlated with the diameter. The velocity angle proved to be an excellent biomarker in the differentiation between volunteers and patients with BAV, BAV morphotypes, and BAV phenotypes, with an area under the curve bigger than 0.90, and higher predictor important scores.

**Conclusions:**

Through the application of a novel 3D quantification method, hemodynamic parameters related to flow direction, such as flow eccentricity, velocity angle, and regurgitation fraction, presented the best relationships with a local diameter and effectively differentiated patients with BAV from healthy volunteers.

## Introduction

Bicuspid aortic valve (BAV) is the most common congenital heart defect (1), with an estimated prevalence between 1 and 2%, increasing in white population and men (3:1) (2). Ascending aorta (AAo) dilation is presented in approximately 50% of patients with BAV, and it is associated with the increased risk of aortic dissection, rupture, and sudden death (3, 4). The most common BAV leaflet fusion phenotypes involve the right-left cusps (BAV-RL), and right-non-coronary cups (BAV-RN), with the prevalence of around 80 and 17% (4, 5), respectively. Genetic, histological, mechanical, and hemodynamic factors related to aortopathy in patients with BAV are still poorly understood (6–9). Currently, preventive aortic surgery is indicated when the diameter of the AAo is larger than 50 [mm] in patients with any of the following risk factors: aortic coarctation, systemic hypertension, a family history of aortic dissection, or rapid aortic growth (>3–5 mm/year) in experienced hands (10). However, these indications are debatable since some aortic events occur with aortic diameters below the suggested threshold (11). Therefore, there is a need for a better understanding of the mechanisms that influence the progression of these structural changes, which may allow the development of prognostic models for risk assessment, the indication of surgical correction, and pre- and post-operative monitoring (12, 13).

Cardiovascular hemodynamic parameters quantified using 4D-flow cardiac magnetic resonance (CMR) are emerging as the essential biomarkers in the early diagnosis of cardiovascular diseases, bringing new insights about complex flows as in patients with BAV (6, 12, 14). Recent studies have provided strong evidence that altered blood flow hemodynamics and wall shear stress (WSS) in the AAo of patients with BAV are associated with histological and proteolytic changes in the aortic wall, which may induce aortic remodeling (6, 9, 15). Moreover, altered WSS has been related to aortic wall disruption (12, 15), and the separated axial (WSSA) and circumferential (WSSC) components of WSS with abnormal flow eccentricity and leaflet fusion phenotype and extent (6, 16–19). Furthermore, several other hemodynamic parameters, such as flow eccentricity, circulation, vorticity, and helicity density, have also been reported in patients with BAV. However, they have mainly been assessed in a limited number of 2D planes (14, 20) with possible unappreciation of important aspects. Some studies have shown the application of three-dimensional (3D) WSS and its association with the valvular dysfunction (21) and the elastic fiber thinning (15), but the WSS quantification has been most often limited to its magnitude, neglecting its axial and circumferential components, at least in 3D applications. Other studies have shown in 3D the relationship between the absolute local normalized helicity (22) and energy loss (23) and the aortic dilation in patients with BAV. Considering many abnormal flow descriptors proposed, it is of utmost importance to identify those deserving special attention for the follow-up of patients with BAV.

A comprehensive methodology can help to identify those parameters related to aortic dilation in patients with BAV. Previously, we have developed a seamless computational framework to obtain several 3D quantitative parameters, which have been validated in phantoms and different cohorts of patients including aortic dissection (24, 25) and transposition of the great arteries (26, 27). This study aimed to compare quantitative 3D hemodynamic parameters between healthy volunteers (HVs) and patients with BAV and their relationships with aortic dilation in clinically relevant subgroups of patients with BAV. We hypothesize that there are differences in hemodynamic parameters between clinically relevant patients with BAV subgroups (morphotypes and phenotypes), such as those with a non-dilated ascending aorta (BAV-Non-AAoD), which includes non-dilated (BAV-NonD) and root dilated (BAV-RootD), with dilation of the AAo (BAV-AAoD), BAV-RL, BAV-RN, and the group of volunteers.

## Methods

### Study Population

A total of 49 patients with BAV-RL and 25 BAV-RN with AAo diameters ≤ 50 mm and no severe valvular disease (aortic regurgitation ≤ III, maximum aortic valve velocity <3 m/s by echocardiography) were consecutively and prospectively recruited between June 2014 and December 2015 at the Hospital Universitari Vall d'Hebron (Barcelona, Spain). Inclusion criteria were as follows: age >18 years, no connective tissue disorders, no aortic coarctation or other congenital heart diseases, no previous aortic surgery or aortic valve replacement, and no contraindication for CMR. A total of 48 HVs matched for sex, weight, height, body surface area (BSA), and stroke volume were also included. The local ethics committee approved the study, and informed consent was obtained from all participants.

### Cardiovascular Magnetic Resonance Protocol

4D-flow CMR data were obtained in a clinical GE 1.5T Signa scanner (GE Healthcare, Waukesha, WI, USA) using the Vastly undersampled Isotropic Projection Reconstruction (VIPR) technique (28, 29). The volumetric acquisition included the entire thoracic aorta and was performed with retrospective ECG-gating during free-breathing and without administration of an endovenous contrast agent. Acquisitions were made using the following parameters: field of view (FOV) 400 mm × 400 mm × 400 mm, voxel size of 2.5 mm × 2.5 mm × 2.5 mm, flip angle 8°, repetition time 4.2–6.4 ms, echo time 1.9–3.7 ms, and velocity encoding 200 cm/s. This dataset was reconstructed with a temporal resolution that ranged between 21–32 ms.

### Aortic Diameters and Aortic Morphotype

The two-dimensional balanced steady-state free precession (bSSFP) cine CMR images were used to assess BAV morphotype and aortic diameters as described (16). Briefly, the three aortic root cusp-to-commissure diameters were measured using double-oblique cine images at the level of the aortic root at end-diastole, and the maximum value was retained for the analysis. Similarly, AAo diameter was measured at end-diastole by double-oblique cine CMR at the level of the pulmonary artery bifurcation. To determine the presence of aortic root or ascending dilation, aortic diameters were adjusted with a logarithm transformation to set the z-score for both sinuses (zsinus) and AAo (zAAo) accounting for sex, age, and BSA as described by Campens et al. (30). Using a z-score cutoff value for the definition of aortic dilation of two standard errors of the estimate, patients were categorized according to the aorta segment predominantly or exclusively involved in dilation according to Della Corte's classification (31). Thus, patients were classified as non-dilated (zsinus ≤ 2 and zAAo ≤ 2), root-dilated morphotype (zsinus>2 and zsinus>zAAo), or AAo-dilated morphotype (zAAo>2 and zAAo>zsinus). Given the small number of patients (seven patients) with rood-dilated morphotype that we have, the groups of patients with BAV were divided only into two morphotypes categories, BAV-Non-AAoD (where we include 18 non-dilated patients and seven root-dilated patients) and BAV-AAoD (where we include the 49 patients with dilatation of the ascending aorta).

### 3D Quantification of Hemodynamic Parameters and Diameter

**Figure 1:**
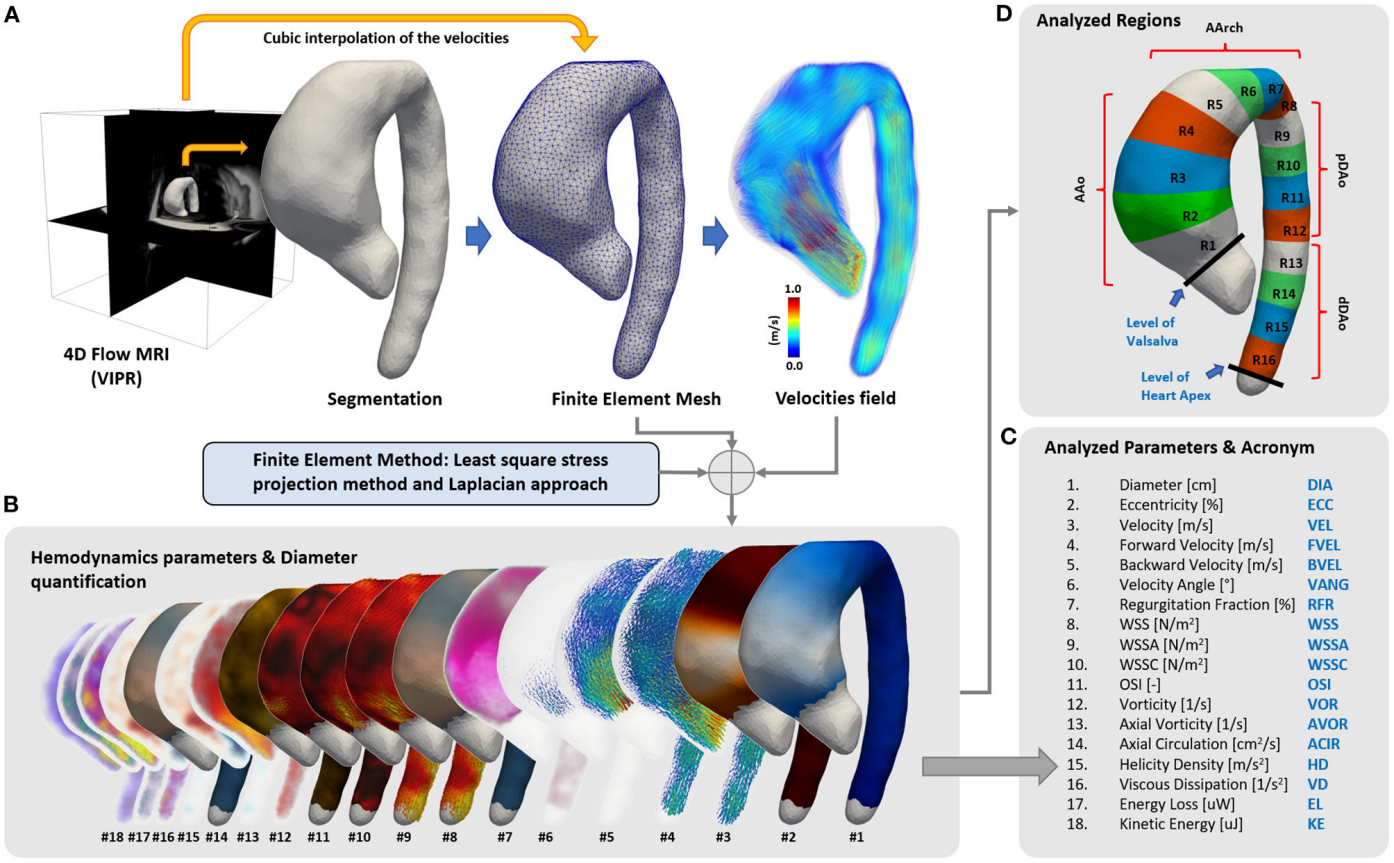
Summary of the quantification process. **(A)** From the 4D-flow CMR acquisition, a semiautomatic segmentation of the aorta was generated and transformed into a tetrahedral mesh. Then, 4D-flow CMR velocity values were interpolated to each node of the mesh, and **(B,C)** 3D maps of 17 hemodynamics parameters and the diameter were calculated using finite elements. **(D)** A total of 16 different regions of the aorta were created to compare the data between volunteers and patients.

The hemodynamic parameters and the diameters in the entire thoracic aorta were obtained based on a finite-element method described previously (32–36) (Figure 1A). To provide a ground for location-specific comparisons, 16 regions (Figure 1D) were semi-automatically selected. Those regions were delimited by anatomical landmarks, regions 1 to 4 = AAo (between the Valsalva level and the brachiocephalic trunk), regions 5 to 8 = aortic arch (AArch, between the brachiocephalic trunk and the isthmus level), regions 9 to 12 = proximal descending aorta (pDAo, between the isthmus level and the level of Valsalva), and regions 13 to 16 = distal descending aorta (dDAo, between the Valsalva level and the diaphragmatic level).

In each region, the mean value of 17 hemodynamic parameters and the aortic diameter were calculated (Figures 1B,C) for each cardiac phase. Peak systolic values were retained for comparison [averaged at peak systole using 1 time-frame before and two time-frames after to reduce noise in the data (16)], except for regurgitation fraction (35) and oscillatory shear index (OSI) calculated along the entire cardiac cycle. The detailed information about the quantification of each parameter is provided in Supplementary Figures S1–S8.

### Statistical Analysis

The global statistical differences between healthy volunteers and patients with BAV were evaluated using a multivariate analysis of variance (MANOVA), with Pillai's trace statistic, whereas the local statistical differences were evaluated using the Mann–Whitney U test. To determine which parameters were more relevant to classify BAV morphotypes and phenotypes with respect to healthy volunteers, the receiver operating characteristic (ROC) curves and the minimum redundancy maximum relevance (MRMR) algorithm were used. Moreover, the Spearman's correlation coefficient was used to find the relationship between the hemodynamic parameters and the maximum AAo diameter for all patients with BAV together and for each dilation morphotype and fusion phenotype separately. The Spearman's correlation values (S) were discretized in three different groups: good (0.5 < |S| < =0.7), strong (0.7 < |S| < =0.9), and excellent (0.9 < |S| < =1). The statistical analysis was performed using SPSS Statistics (version 25.0 IBM SPSS, Chicago, IL).

## Results

### Demographics

A total of 74 patients with BAV (43 men, age 45[37–61] years) and 48 HV (29 men, age 41[30–49] years) were included in the study. Demographic and clinical variables of patients with BAV and HV are shown in Table 1. HVs were matched to patients with BAV in terms of sex, weight, height, BSA, and stroke volume. RL fusion phenotype was present in 66% of patients with BAV. According to the dilation morphotype, 34% patients with BAV (7 BAV root-dilated and 18 BAV non-dilated, or 19 BAV-RL and 6 BAV-RN) were classified as non-ascending aorta dilated (BAV-Non-AAoD) and 66% patients with BAV (19 BAV-RN and 30 BAV-RL) as AAo-dilated (BAV-AAoD).

**Table 1:** Volunteer and patient demographics.

	**Volunteers**	**BAV-patients**	***p*-value**
	**Median [IQR]**	**Median [IQR]**	
Male/female (male%)	29/19(60%)	43/31(72%)	0.470^†^
Age (years)	41 [30–49]	45 [37–61]	0.008^*^
Weight (Kg)	73.0[64.8–80.0]	73.5[61.8–80.0]	0.856
Height (cm)	169.5[163.0–175.0]	170.0[163.0–178.0]	0.929
BSA (m^2^)	1.9[1.7–1.9]	1.9[1.7–2.0]	0.953
Stroke volume (ml)	88.1[77.5–114.6]	92.9[68.9–111.1]	0.805
Ejection fraction (%)	66.6[62.6–69.7]	59.4[55.2–63.6]	0.000^*^
Phenotype	–	49RL, 25RN	–
Morphotype	–	25 Non-AAo-D (18 Non-D and 7 Root-D), 49 AAo-D	–
Hypertension	–	30(No), 44(Yes)	–

* *Statistically significant differences (p < 0.05);*

† *Chi-square test*.

### Hemodynamic Parameters and Diameter vs. Aortic Morphotypes and Phenotypes

**Figure 2:**
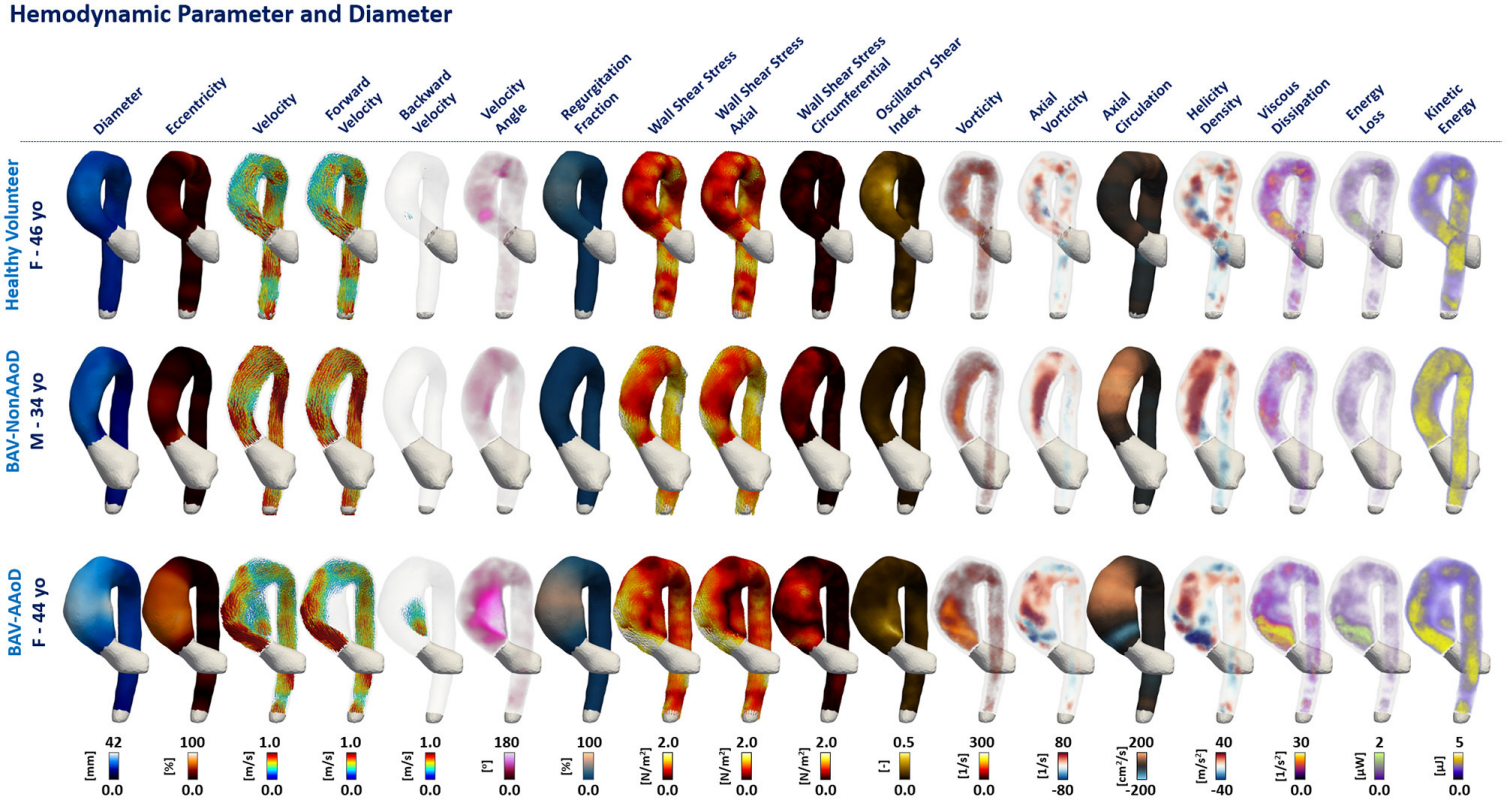
Three-dimensional maps of a representative HV and patients with BAV. Columns represent each analyzed parameter (Figure 1C). First row shows a HV, second row a non-dilated BAV (BAV-Non-AAoD) patient, and third row a patient with BAV with ascending aorta dilation (BAV-AAoD).

The three-dimensional maps of the different parameters are shown in Figure 2 for one representative volunteer and two representative patients (one BAV-Non-AAoD and one BAV-AAoD). The phenotype was not included in this figure due to the limitation of selecting a representative morphotype case in each phenotype group. Differences between volunteers and patients with BAV in the AAo and part of the AArch are visible for several parameters: diameter, eccentricity, backward velocity, velocity angle, regurgitation fraction, WSSC, and axial circulation.

**Figure 3:**
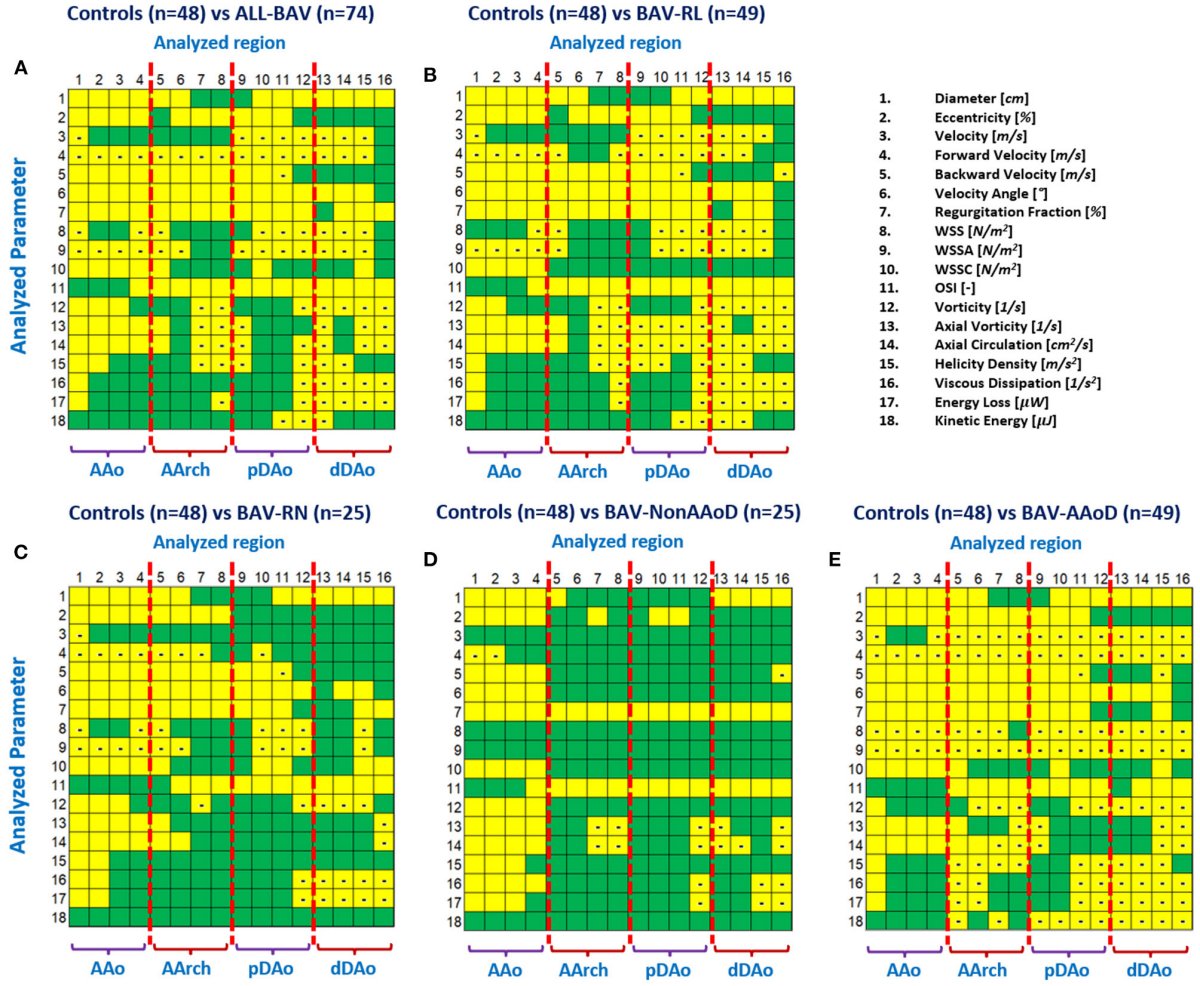
Statistical differences between groups: **(A)** HV vs. all BAV; **(B)** HV vs. BAV-RL; **(C)** HV vs. BAV-RN; **(D)** HV vs. BAV-Non-AAoD; **(E)** HV vs. BAV-AAoD. Yellow marks highlight statistically significant differences between compared groups (*p*-value <0.05). The minus sign in yellow boxes indicates the value was lower in BAV than in volunteers.

From the multivariate analysis test MANOVA, we obtained *p*-values from Pillai's trace of <0.001 for the HV vs. BAV-All, HV vs. BAV-Non-AAoD, HV vs. BAV-AAoD, HV vs. BAV-RL, and HV vs. BAV-RN. This test showed significant differences between the HV and all BAV groups in all parameters tested. In Figure 3, the comparison between the different BAV groups and HV is shown for each parameter (rows) and aortic region (columns). Most of the analyzed parameters showed significantly higher values in patients with BAV than HV in the AAo and the proximal AArch (regions 1 to 6). In contrast, in the DAo, most of the significant differences resulted from lower values in BAV groups. Eccentricity, backward velocity, velocity angle, regurgitation fraction, WSSC, vorticity, axial vorticity, and axial circulation were consistently greater in patients with BAV than in HV, even in the absence of clinically significant dilation. All parameters (except velocity) showed significant differences in more than one segment for almost all analyzed cases. Velocity, forward velocity, WSS, and WSSA showed lower values in patients with BAV compared to HV.

We also observed similar results for fusion phenotypes (Figures 3B,C) compared to HV. We found that the significant differences in patients with BAV-RN are more concentrated between the ascending aorta and aortic arch, in comparison with BAV-RL, probably, this is influenced by the dilation of the aorta at the proximal part of the aortic arch in patients with BAV-RL, and the descending aorta of these patients show only a few parameters with significant differences. The velocity angle and regurgitation fraction were significantly different for most regions in BAV-RN and BAV-RL. Nevertheless, BAV-RL showed more differences for parameters related to the flow turbulence (parameters from 12 to 17) in the descending aorta with lower values for patients with BAV.

In Supplementary Figure S9, we evaluated the differences between morphotypes and phenotypes groups. BAV-AAoD presented significant differences compared to the BAV-Non-AAoD for velocity, forward velocity, velocity angle, WSS, WSSA, and kinetic energy in the entire aorta. Other parameters such as backward velocity, regurgitation fraction, and vorticity are mostly significant in the ascending aorta. Interestingly, some parameters, which are related to the rotation of the flow, are only significant in the proximal part of the aortic arch, as axial circulation, helicity density, viscous dissipation, and energy loss. Comparing BAV-RL and RN phenotypes groups, we found significant differences in velocity angle, axial vorticity, and axial circulation in more than one region.

Table 2 shows the mean and standard deviation values of each parameter in the AAo (averaged between the regions 1 to 4) for patients with HV and BAV. Patients with BAV presented higher values in most of the parameters in comparison with HV group, whereas velocity, forward velocity, WSS, WSSA, and kinetic energy were lower in patients with BAV. In the AArch, pDAo, and dDAo, the velocity, forward velocity, WSS, WSSA, and the parameters related to the turbulence such as vorticity, axial vorticity, axial circulation, helicity density, viscous dissipation, energy loss, and kinetic energy were lower than in volunteers (Supplementary Tables S1–S3).

**Table 2:** Mean and (standard deviation) for each parameter in the AAo section (regions 1 to 4).

**Parameter**	**Volunteer**	**BAV**	**BAV**	**BAV**	**BAV**	**BAV**
		**ALL**	**RN**	**RL**	**AAoD**	**Non-AAoD**
Diameter [cm]	2.87(0.39)	4.06(0.70)	4.04(0.73)	4.10(0.63)	4.34(0.60)	3.50(0.53)
Eccentricity [%]	18.76(8.08)	41.02(13.56)	40.26(14.20)	42.51(12.14)	42.65(13.02)	37.83(14.10)
Velocity [m/s]	0.41(0.14)	**0.39(0.11)** *	**0.38(0.11)** *	**0.39(0.11)** *	**0.36(0.11)** *	0.43(0.10)
Forward velocity [m/s]	0.39(0.14)	**0.26(0.10)** *	**0.27(0.11)** *	**0.24(0.08)** *	**0.22(0.07)** *	**0.33(0.11)** *
Backward velocity [m/s]	0.00(0.00)	0.03(0.02)	0.03(0.02)	0.03(0.03)	0.03(0.02)	0.02(0.02)
Velocity angle [°]	17.79(7.10)	51.05(15.91)	48.54(16.67)	55.97(13.03)	56.24(12.90)	40.88(16.38)
Regurgitation fraction [%]	13.37(8.46)	41.50(21.81)	41.73(25.35)	41.05(12.28)	44.09(15.34)	36.43(30.23)
WSS [N/m^2^]	0.64(0.25)	**0.55(0.21)** *	**0.55(0.22)** *	**0.55(0.21)** *	**0.50(0.19)** *	0.65(0.22)
WSSA [N/m^2^]	0.60(0.26)	**0.41(0.18)** *	**0.42(0.19)** *	**0.38(0.16)** *	**0.35(0.14)** *	**0.52(0.20)** *
WSSC [N/m^2^]	0.14(0.06)	0.29(0.14)	0.27(0.12)	0.31(0.17)	0.28(0.14)	0.29(0.13)
OSI [–]	0.17(0.05)	0.17(0.04)	0.17(0.04)	0.17(0.04)	**0.16(0.04)** *	0.18(0.05)
Vorticity [1/s]	62.76(17.72)	72.17(23.24)	70.40(23.20)	75.65(23.04)	68.36(23.43)	79.65(21.05)
Axial vorticity [1/s]	3.30(9.47)	17.38(12.90)	17.86(10.15)	16.45(17.08)	16.48(11.14)	19.14(15.70)
Axial circulation [cm^2^/s]	27.18(64.00)	240.86(163.24)	238.18(128.90)	246.11(215.96)	259.67(170.06)	204.00(142.72)
Helicity density [m/s^2^]	8.18(4.47)	9.70(5.40)	9.22(4.56)	10.63(6.66)	9.33(5.78)	10.43(4.48)
Viscous dissipation [1^3^/s^2^]	6.25(3.36)	7.66(4.22)	7.29(3.95)	8.37(4.64)	7.34(4.38)	8.28(3.84)
Energy loss [uW]	0.39(0.22)	0.47(0.27)	0.45(0.26)	0.51(0.30)	0.46(0.28)	0.50(0.26)
Kinetic energy [uJ]	2.01(1.34)	**1.84(0.97)** *	**1.84(0.98)** *	**1.82(0.94)** *	**1.68(0.89)** *	2.15(1.04)

The mark (*) and bold numbers shows if the parameter is lower in the BAV than volunteer groups.

### Analysis of ROC Curves and MRMR Algorithm

**Figure 4:**
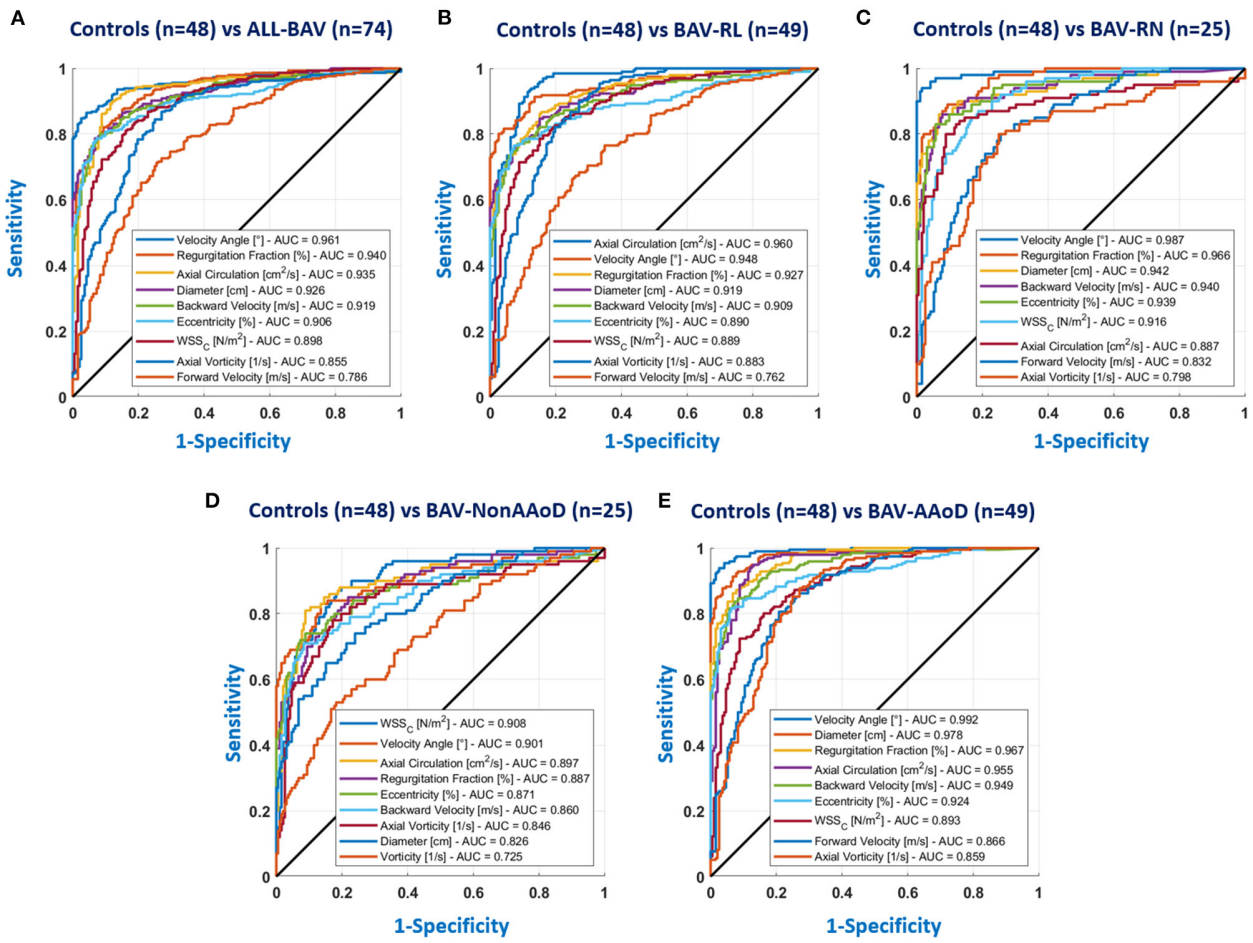
ROC curves for the AAo; **(A)** HV vs. all BAV; **(B)** HV vs. BAV-RL; **(C)** HV vs. BAV-RN; **(D)** HV vs. BAV-Non-AAoD; **(E)** HV vs. BAV-AAoD. The parameters of the legend are the nine parameters with bigger area under the curve (AUC) values.

To identify the capacity of each parameter to differentiate between BAV and HV, receiver operating characteristic (ROC) curves were calculated. In the AAo, the area under the ROC curves (Supplementary data online Supplementary Table S4) was >0.8 for diameter, eccentricity, backward velocity, velocity angle, regurgitation fraction, WSSC, axial vorticity, and axial circulation (refer to Figure 4). Of note, the velocity angle was the best performing parameter, showing a specificity close to 99% to classify BAV-AAoD from volunteers, which was reduced to 90% in patients with BAV-Non-AAoD. Similar performances were obtained for eccentricity, backward velocity, WSSC, and axial circulation. In the other aortic sections (AArch, pDAo, and dDAo), only one parameter showed an area under the ROC curves >0.8 (Supplementary data online Supplementary Tables S5–S7), the most characteristic being the regurgitation fraction in the AArch sections.

In Supplementary Figure S10, we show the ROC curves for the AAo between morphotypes and phenotypes groups. For patients with BAV-RN vs. BAV-RL, we obtained AUC values lower than 0.63, being the velocity angle the greater value. These results are in concordance with the few significant differences found in Supplementary Figure S9A. Comparing BAV-Non-AAoD vs. BAV-AAoD, excluding diameter and forward velocity, whose values are attributed to the greater area of the segment, the parameter with the highest AUC (0.77) was also the velocity angle.

The MRMR algorithm was applied in each section (AAo, AArch, pDAo, and dDAo), to assess similarities with the ROC curves. In the AAo, the velocity angle, axial circulation, regurgitation fraction, diameter, eccentricity, circumferential WSS, backward velocity, forward velocity, and axial vorticity were the parameters with a greater importance predictor score for most of the cases (HV vs. BAV-All, HV vs. BAV-RL, HV vs. BAV-RN, and HV vs. BAV-AAoD). For HV vs. BAV-Non-AAoD, the velocity angle was the parameter with a greater importance predictor score (Supplementary data online Supplementary Figure S11; Supplementary Table S8). Additionally, in Supplementary data online Supplementary Tables S9–11, we show the predictor important scores for the AArch, pDAo, and dDAo, respectively.

The MRMR was also evaluated between morphotypes and phenotypes only for the AAo (Supplementary Figure S12). The comparison of BAV-RN and BAV-RL shows only three parameters with a higher importance prediction score (vorticity, axial vorticity, and axial circulation), and two of them are in concordance with the parameter found in the Supplementary Figure S9A. For BAV-Non-AAoD and BAV-AAoD, we found that the diameter, backward velocity, WSS, forward velocity, regurgitation fraction, velocity angle, vorticity, and axial vorticity were the parameters with a higher importance prediction score.

### Correlation Matrices of the Hemodynamic Parameters

**Figure 5:**
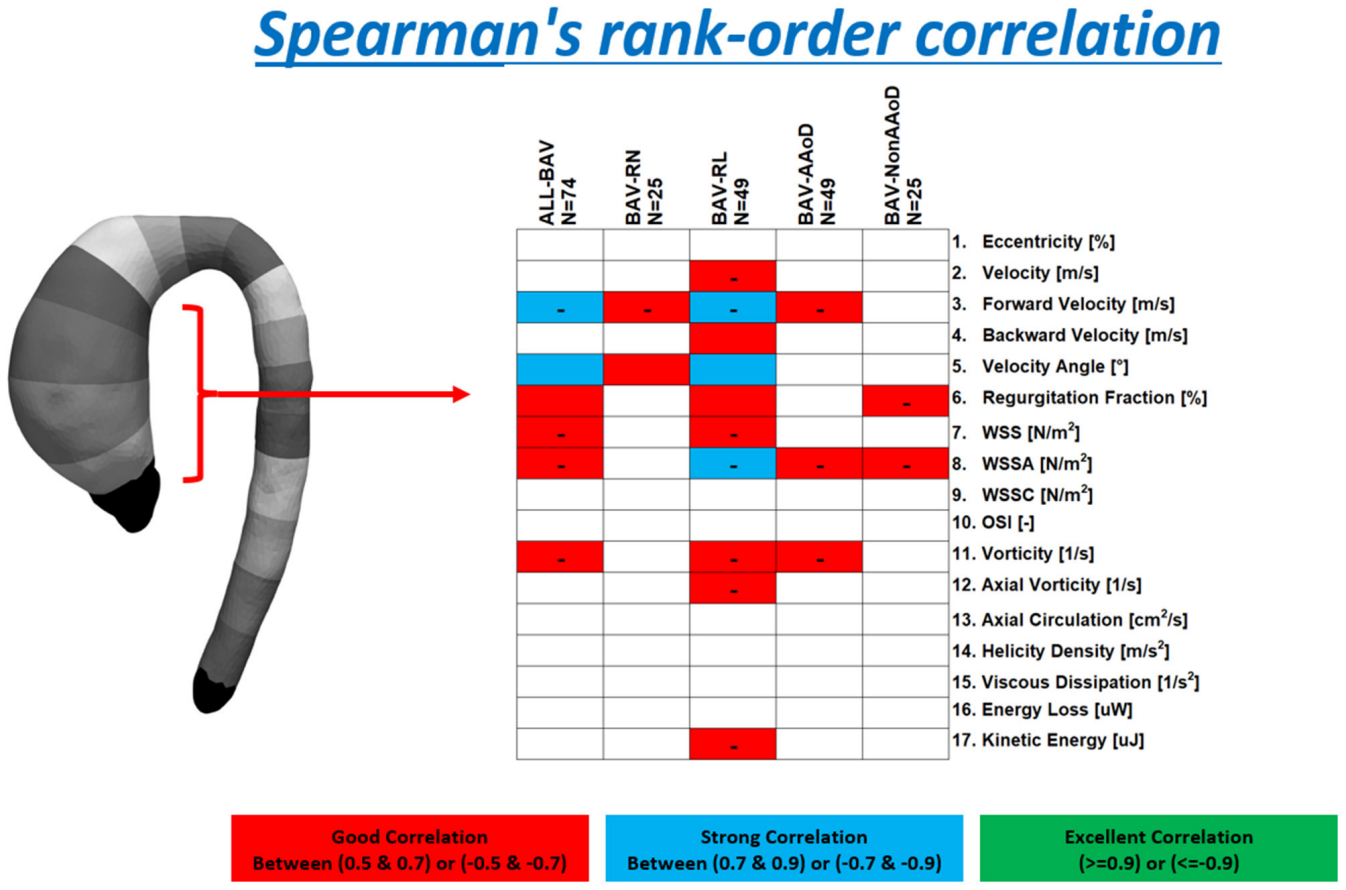
Spearman's correlation coefficient values for patients with BAV, BAV-RN, BAV-RL, BAV-AAoD, and BAV-Non-AAoD are reported. These values were grouped as good correlation (red box, Spearman's correlation between |0.5| and |0.7|), strong correlation (blue box, Spearman's correlation between |0.7| and |0.9|), and excellent correlation (green box, Spearman's correlation above |0.9|). The minus sign indicates a negative correlation.

In Figure 5, Spearman's correlation coefficient values between the diameter and each hemodynamic parameter in the AAo (the value was averaged between regions 1 to 4) are shown for patients with BAV. Considering all patients with AV together, the aortic diameter presented a strong correlation with forward velocity and velocity angle, the regurgitation fraction, WSS, WSSA, and vorticity presented a good correlation with the diameter. For patients with BAV-RN showed a good correlation for forward velocity and velocity angle. Patients with BAV-RL showed more parameters correlated with the diameter that the other cases, the forward velocity, velocity angle, and WSSA, which are correlated strongly with the diameter, the velocity, backward velocity, regurgitation fraction, WSS, vorticity, axial vorticity, and kinetic energy, have a good correlation with the diameter in this group. For patients with BAV-AAoD, we found a good correlation between the diameter and the forward velocity, WSSA, and vorticity. Finally, patients with BAV-Non-AAoD showed a good correlation for the regurgitation fraction and WSSA.

## Discussion

One of the strengths of our study is that it provides a comprehensive analysis of a novel quantification tool to obtain several 3D aortic maps of different hemodynamic parameters in a cohort of patients with BAV. Moreover, we presented an extensive comparison with HV, identifying a subset of high-performing parameters. Several hemodynamic parameters showed significant differences between HV and patients with BAV in the AAo. Among them, eccentricity, backward velocity, velocity angle, regurgitation fraction, WSSC, axial vorticity, and axial circulation showed the most marked differences, even in patients without aortic dilation.

**Figure 6:**
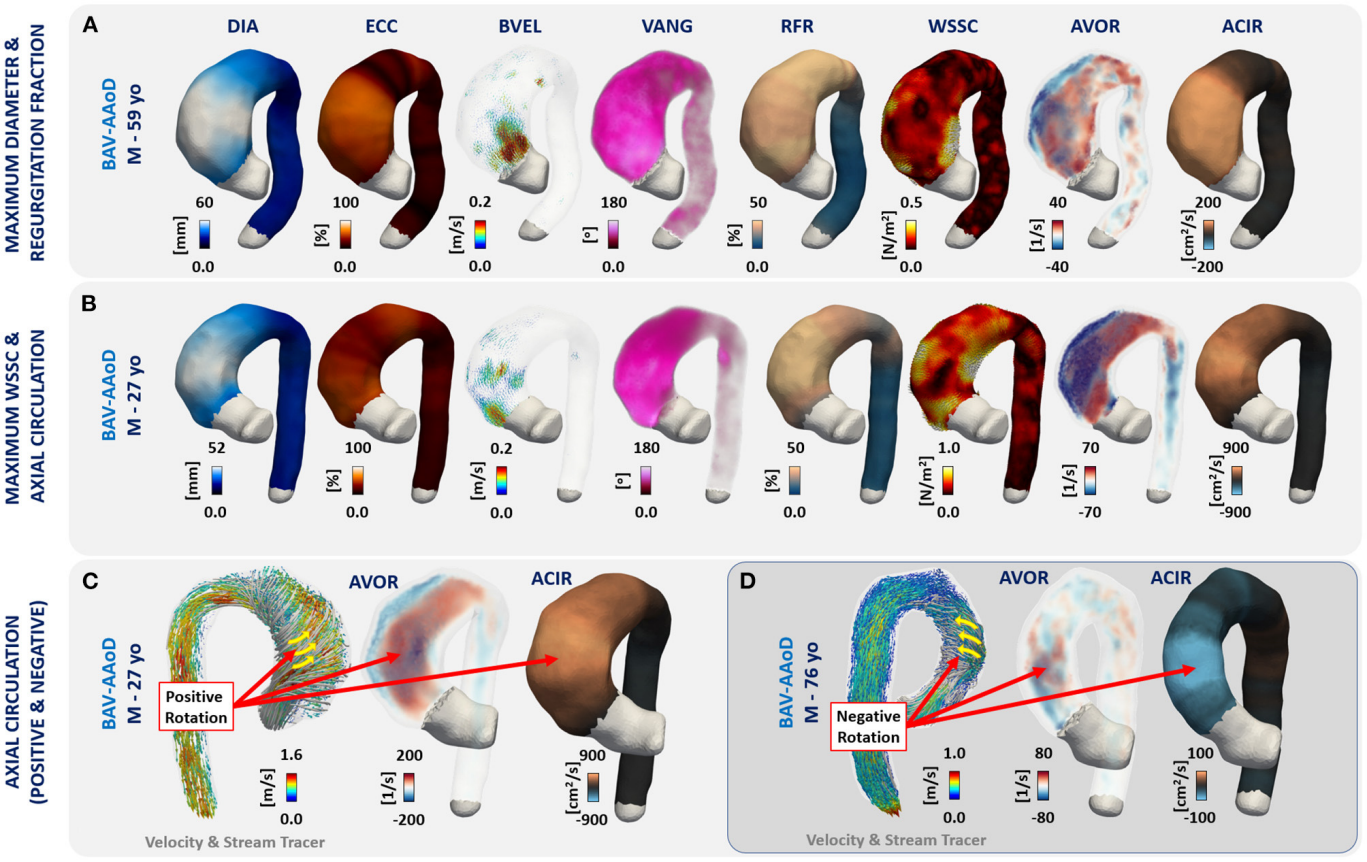
**(A)** A patient with maximum values of diameter and regurgitation fraction; **(B)** a patient with maximum WSS_C_ and axial circulation; **(C)** a patient with positive axial circulation; and **(D)** one case with negative axial circulation.

In patients with BAV with AAo dilation, eccentricity, backward velocity, velocity angle, regurgitation fraction, WSSC, axial vorticity, and axial circulation showed statistical differences in the AAo compared to volunteers, with higher values in patients with BAV. Moreover, the velocity, forward velocity, WSS, and WSSA also showed statistical differences between patients with BAV-AAoD and volunteers, but lower values characterized the v with BAV. The WSSC was the only present in the AAo of patients with BAV with AAo dilation and may thus arise from dilation *per se* (17, 37). Some of these parameters have been previously studied separately from a qualitative point of view (38, 39). Those studies have concluded that the altered jet direction (i.e., flow eccentricity) is one of the major contributors to aortic dilation in patients with BAV and related to axial circulation (refer to Figure 6). Flow eccentricity in patients with BAV (40) may alter the integrity of the aortic wall and promote dilation of the AAo (7, 41).

Wall shear stress has also been extensively studied in patients with BAV (6, 9, 15–17, 21, 39, 42, 43). In this study, we found statistically significant differences for the magnitude of WSS between HV and patients with BAV with AAo dilated and all patients together. Interestingly, the WSS was not significant in patients with BAV-Non-AAoD. The WSSC showed larger values in BAV and significant differences in the AAo (Figure 3) in comparison with HV, even in non-dilated patients. Similar results have been reported in the literature (9, 44, 45).

To the best of our knowledge, the velocity angle and the axial circulation in 3D domain have not been previously studied in a large cohort of patients with BAV. The velocity angle represents the deviation of the flow velocity at each point in the vessel's lumen concerning their axial direction calculated with the Laplace equation (32) (refer to Supplementary Figure S4). The velocity angle also showed statistical differences between patients with BAV and HV. Other studies reported similar results when analyzing the direction of flow at the level of the aortic valve (1, 46–48). The circulation represents the integral of vorticity with respect to the cross-sectional area of the vessel, in our case, this vorticity was the axial vorticity at each point of the level set generated with the Laplace equation (32), and for this reason, we call it axial circulation (refer to Supplementary Figure S7 and the reference 36). This parameter showed statistical differences between BAV groups and HV, with higher values in the ascending aorta, bigger than 200 cm^2^/s for patients with BAV and <30 cm^2^/s for HV.

For phenotypes, BAV-RN showed a more significant difference in the proximal part of the aortic arch, probably influenced by the dilation of the vessel in this location (17). In the descending aorta, the BAV-RL showed more significant differences for parameters related to the flow turbulence such as vorticity, axial vorticity, axial circulation, helicity density, viscous dissipation, energy loss, and kinetic energy in the descending aorta with lower values for patients with BAV. Finally, the velocity angle and regurgitation fraction were the most significant for all regions.

Receiver operating characteristic curve analysis showed that in the AAo (Figure 4; Supplementary Table 4), eccentricity, backward velocity, velocity angle, regurgitation fraction, WSSC, axial vorticity, and axial circulation were the best performing parameters in the differentiation between volunteers and patients with BAV, with values under the curve bigger than 0.8. Interestingly, this value was equal to 0.96 for the velocity angle with a sensitivity and specificity of 90%, the largest among all parameters. We validated these results with the application of the MRMR classification algorithm, where the velocity angle, axial circulation, regurgitation fraction, diameter, eccentricity, circumferential WSS, backward velocity, forward velocity, and axial vorticity are the parameters with a bigger importance predictor score for most of the cases (refer to Supplementary Figures S11, S12; Supplementary Tables S8–S11). One interesting parameter analyzed in this study is the axial circulation, which can provide important information on the helical rotation of the blood flow in the entire vessel. Figure 6 shows one representative patient with BAV with positive (men 27 years, Figure 6C) and one with negative (men 76 years, Figure 6D) rotations of the blood flow in the ascending aorta.

Although many parameters were found to be different between HV and patients with BAV, only a few of them showed a good correlation with the diameter of the aorta. The diameter correlated with the forward velocity, WSSA, and vorticity in patients with AAo dilation. There was a strong inverse correlation between the diameter and the forward velocity for all BAV and patients with BAV-RL, and also, there was a strong correlation between the diameter and the velocity angle for the same groups. BAV-RL showed more parameters correlated with the diameter than all other cases.

### Limitations

The main limitation of this study is its cross-sectional nature, which implies that no causal relationship could be inferred. Longitudinal studies are needed to confirm the present results. Another limitation relates to the limited sample size for the group of BAV-RootD, which was included in the non-dilated group, because statistical tests could not be performed on only seven patients. Furthermore, averaging parameters along the circumference of each region can induce sub-estimation of local value. Moreover, the movement of the aorta along the cardiac cycle was not considered in this study since technical limitations in 4D-flow CMR acquisitions (poor contrast and low signal-to-noise ratio) make it difficult to obtain a time-resolved segmentation of the aorta. For that reason, we only analyze the peak systolic cardiac phase. Another limitation of the proposed methodology is that the Laplace approach can be evaluated only for one vessel of interest without branches. Nonetheless, this limitation may also be present in centerline-based methods. The analysis of a large number of parameters can be overwhelming, and for that reason in future contributions, we may generate some artificial intelligence (AI) algorithms that can help to classify groups and categories of these parameters and thus be able to deliver only the parameters that are relevant for a particular pathology, but for this purpose, balanced groups will be needed.

## Conclusion

Through a novel 3D quantification method, a limited number of hemodynamic parameters that differentiate between healthy volunteers and patients with BAV were unveiled and ranked. Those related to the direction of blood flow (forward velocity, velocity angle, regurgitation fraction, and WSSA) presented the best relationships with local diameter; however, eccentricity, backward velocity, velocity angle, regurgitation fraction, WSSC, axial vorticity, and axial circulation are the parameters that accurately differentiate between patients with BAV and volunteers. Longitudinal studies are required to assess whether these parameters are the significant contributors to aortic dilation in patients with BAV.

## Data Availability Statement

The data analyzed in this study is subject to the following licenses/restrictions. The data sets generated during the current study will be made available with prior permission from the partner institutions, the toolbox used to process de data is free available on (https://github.com/JulioSoteloParraguez/4D-Flow-Matlab-Toolbox). Requests to access these datasets should be directed to JS, julio.sotelo@uv.cl.

## Ethics Statement

The studies involving human participants were reviewed and approved by the Local Ethics Committee (Hospital Universitari Vall d'Hebron). The patients/participants provided their written informed consent to participate in this study.

## Author Contributions

JS: responsible for the study concepts, design, and analysis. AG, LD-S, AR-M, AE, and JR-P: data acquisition and clinical interpretation of the data. JS, PF, HM, JM, DH, and SU: technical interpretation of the data. All authors contributed to the article and approved the submitted version.

## Funding

This work was funded by ANID – Millennium Science Initiative Program – ICN2021_004 and ANID – Millennium Science Initiative Program – NCN17_129, CONICYT-FONDECYT Postdoctorado #3170737, ANID – FONDECYT Postdoctorado #3220266, ANID Ph. D. Scholarship 21170592, ANID FONDECYT de Iniciación en Investigación #11200481, ANID FONDECYT #1181057, ANID Ph. D. Scholarship 21180391, the Spanish Society of Cardiology (SEC/FEC-INV-CLI 20/015) and the Biomedical Research Networking Center on Cardiovascular Diseases (CIBERCV). AG has received funding from the Spanish Ministry of Science, Innovation and Universities (IJC2018-037349-I).

## Conflict of Interest

The authors declare that the research was conducted in the absence of any commercial or financial relationships that could be construed as a potential conflict of interest.

## Publisher's Note

All claims expressed in this article are solely those of the authors and do not necessarily represent those of their affiliated organizations, or those of the publisher, the editors and the reviewers. Any product that may be evaluated in this article, or claim that may be made by its manufacturer, is not guaranteed or endorsed by the publisher.
