# Supplementary material for: Fully Three-Dimensional Hemodynamic Characterization of Altered Blood Flow in Bicuspid Aortic Valve Patients With Respect to Aortic Dilatation: A Finite Element Approach
Source: Front Cardiovasc Med. 2022 May 18;9:885338. doi: 10.3389/fcvm.2022.885338 (PMC9157575; doi:10.3389/fcvm.2022.885338)
Supplement: Supplementary file 1 [file Data_Sheet_1.DOCX]

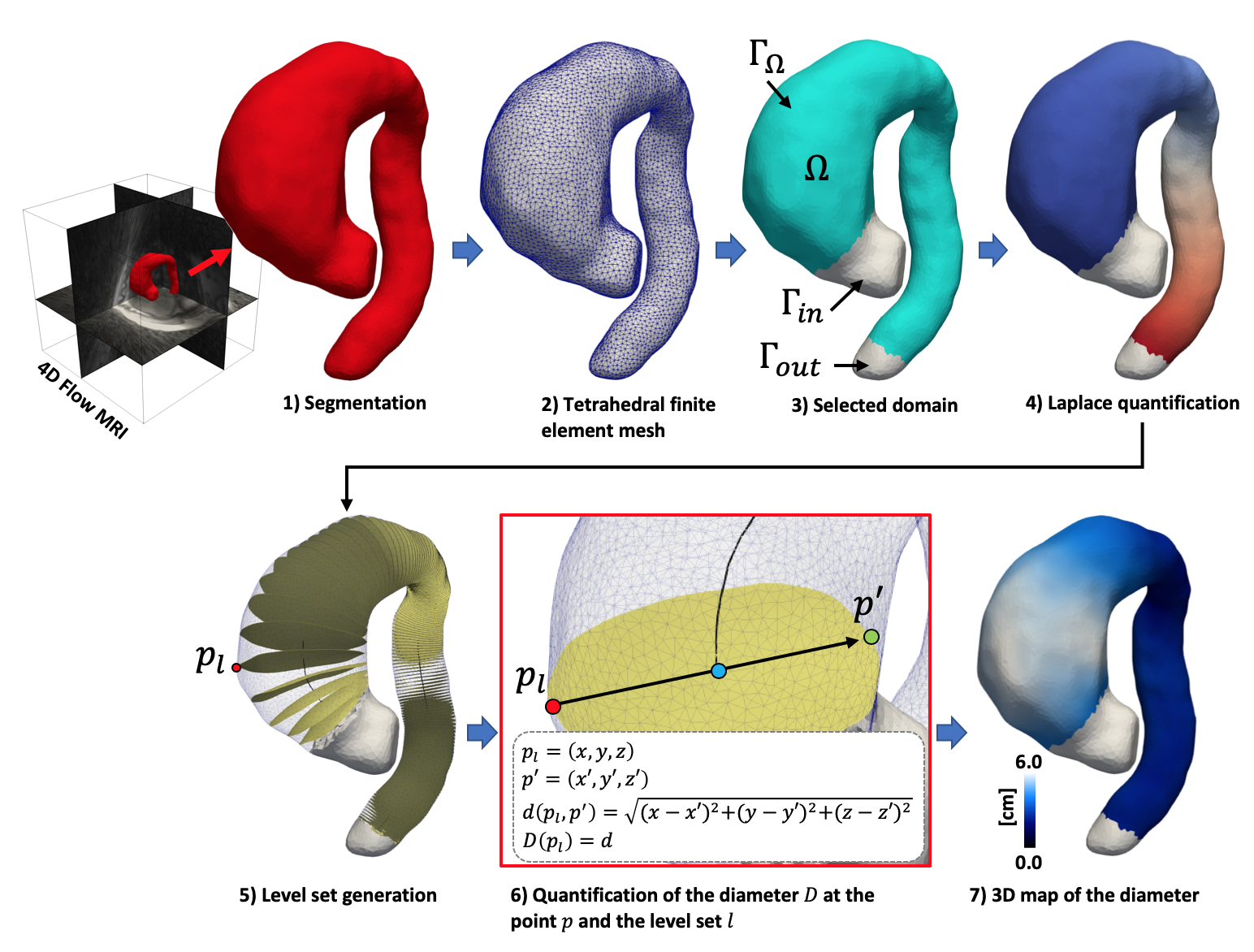


**Supplementary Figure 1.** The diameter is calculated for each node of the vessel surface $\Gamma_{\Omega}$, using our Laplacian finite element approach. Step 1: Segmentation of the vessel of interest from the 4D flow MRI data. Step 2: Generation of the tetrahedral finite element mesh. Step 3: Boundary conditions applications. Step 4: Quantification of our Laplacian solution. Step 5: Using the Laplacian solution we generate one level set for each node of the surface and the diameter was calculated using the equation described in (Step 6). Step 7: Three-dimensional map of diameter.


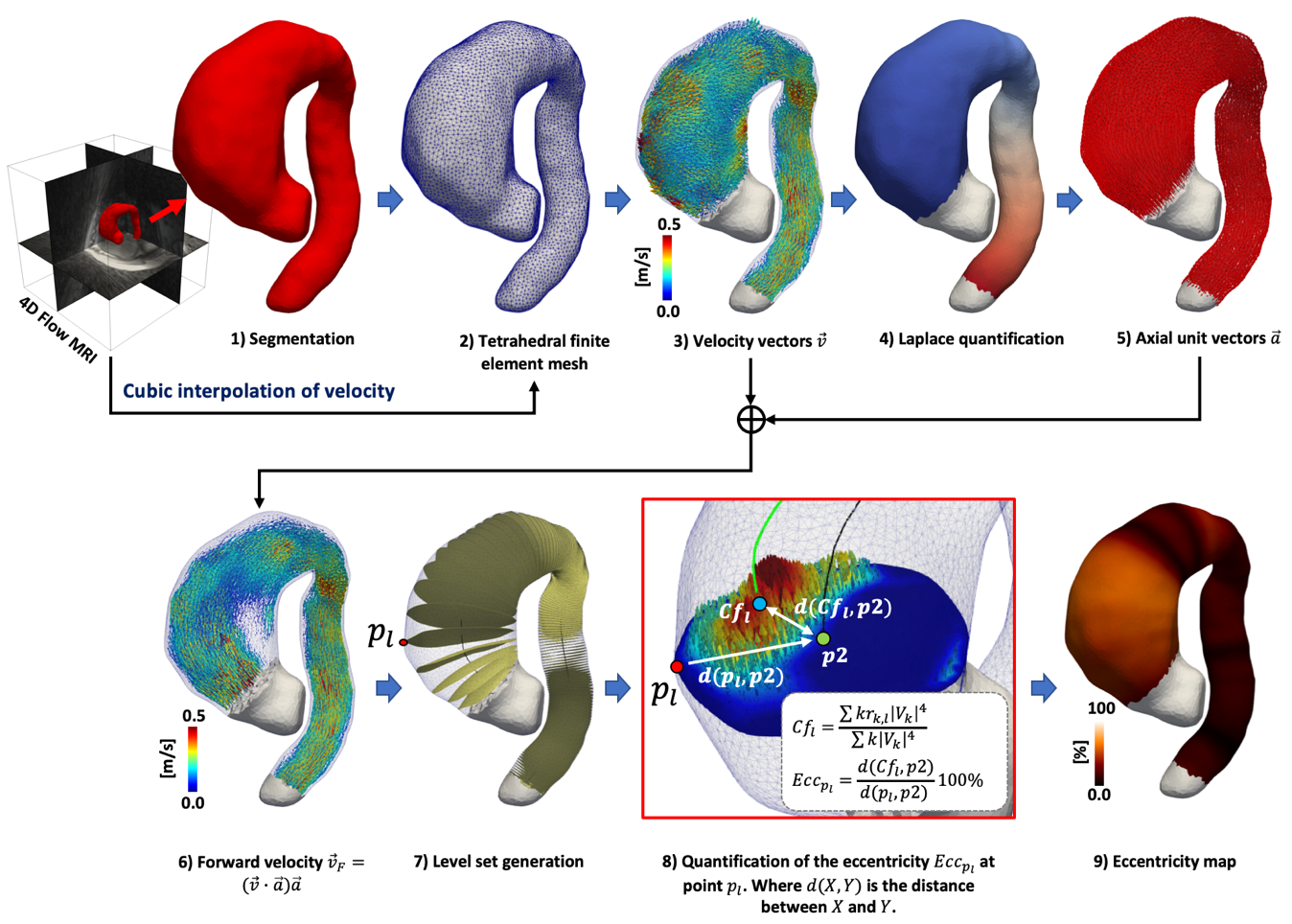


**Supplementary Figure 2.** The eccentricity was calculated as the distance between vessel centerline and the centerline generated by the main flow direction using the forward velocity computed with our Laplacian finite element approach. Step 1: Segmentation of the vessel of interest from the 4D flow MRI data. Step 2: Generation of the tetrahedral finite element mesh. Step 3: Interpolation of the velocity to the tetrahedral finite element mesh. Step 4: Quantification of our Laplacian solution using the same boundary condition (Figure 1 Step 3). Step 5: Axial unit vector quantification, calculated as the normalized gradient of the Laplacian solution. Step 6: Projection of the velocity in the axial direction. Step 7: Level set generated by the Laplacian solution. Step 8: Quantification of the eccentricity, using the axial projection of velocity in each level set. Step 9: Three-dimensional map of eccentricity.


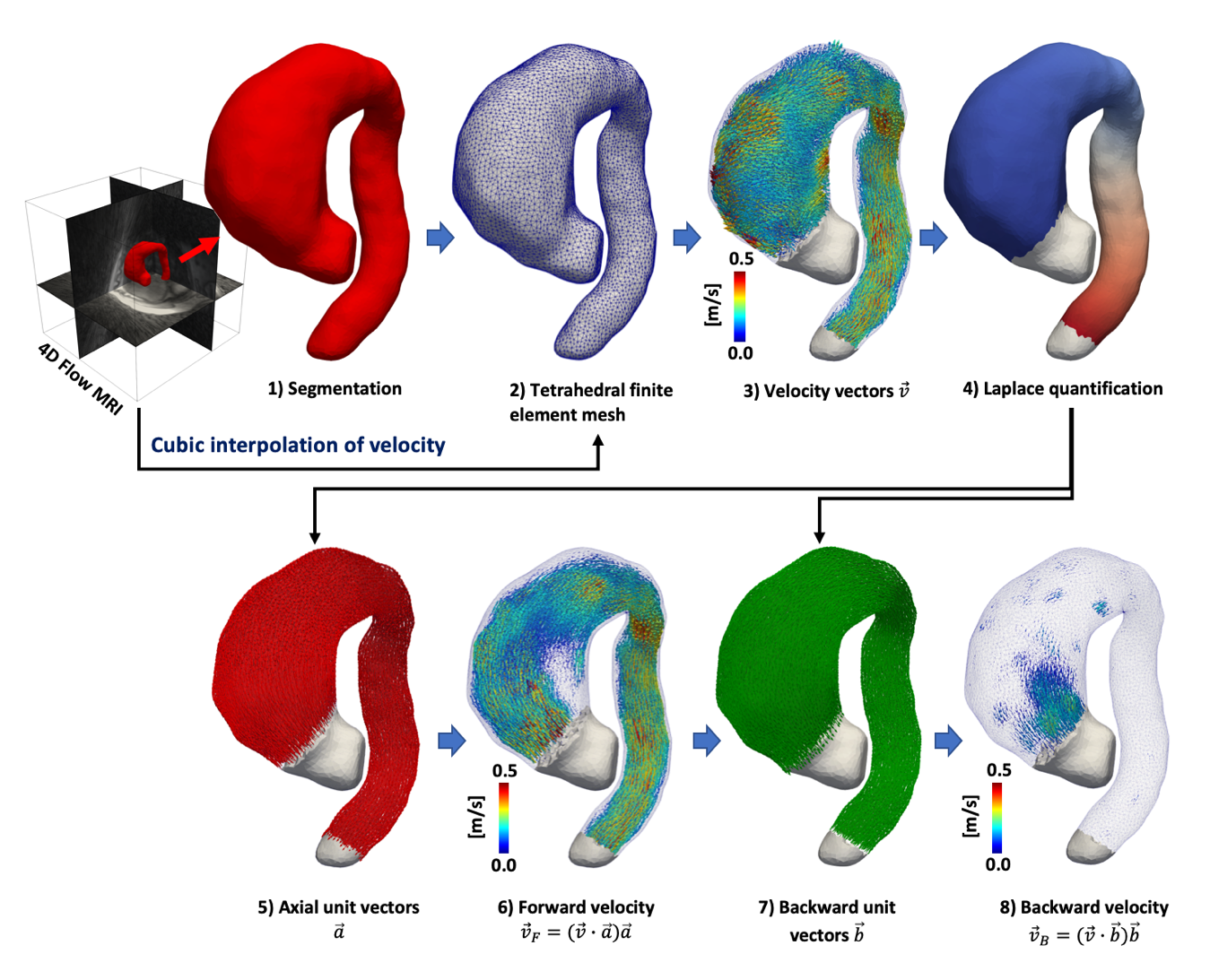


**Supplementary Figure 3.** Forward and backward velocities were obtained by projecting the vector field over the forward and backward unit vector, respectively, using our Laplacian approach based on finite elements. Step 1: Segmentation of the vessel of interest from the 4D flow MRI data. Step 2: Generation of the tetrahedral finite element mesh. Step 3: Interpolation of the velocity to the tetrahedral finite element mesh. Step 4: Quantification of our Laplacian solution using the same boundary condition (Figure 1 Step 3). Step 5: Axial unit vector quantification, calculated as the normalized gradient of the Laplacian solution. Step 6: Projection of the velocity in the forward direction using the axial unit vectors. Step 7: Axial unit vector multiplied by -1 (Backward unit vectors). Step 8: Projection of the velocity in the backward direction using the backward unit vectors.


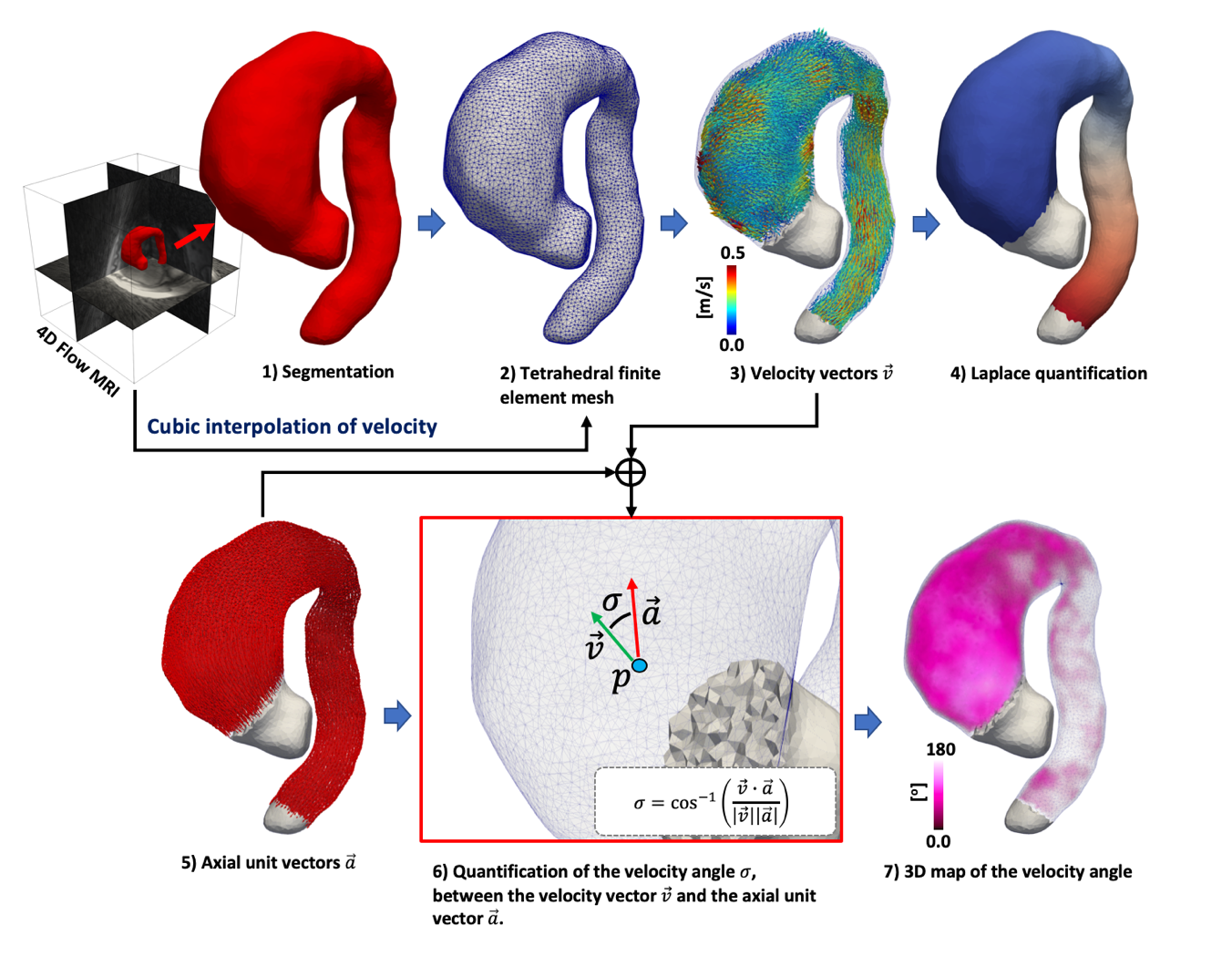


**Supplementary Figure 4.** Angle measured between the velocity vectors and the axial unit vector, in each point of the lumen of the vessel (see $\Omega$ in Figure 1 Step 3). Step 1: Segmentation of the vessel of interest from the 4D flow MRI data. Step 2: Generation of the tetrahedral finite element mesh. Step 3: Interpolation of the velocity to the tetrahedral finite element mesh. Step 4: Quantification of our Laplacian solution using the same boundary condition (Figure 1 Step 3). Step 5: Axial unit vector quantification, calculated as the normalized gradient of the Laplacian solution. Step 6: Quantification of the velocity angle ($\sigma$) for each node of the lumen of the vessel. Step 7: Three-dimensional map of velocity angle.


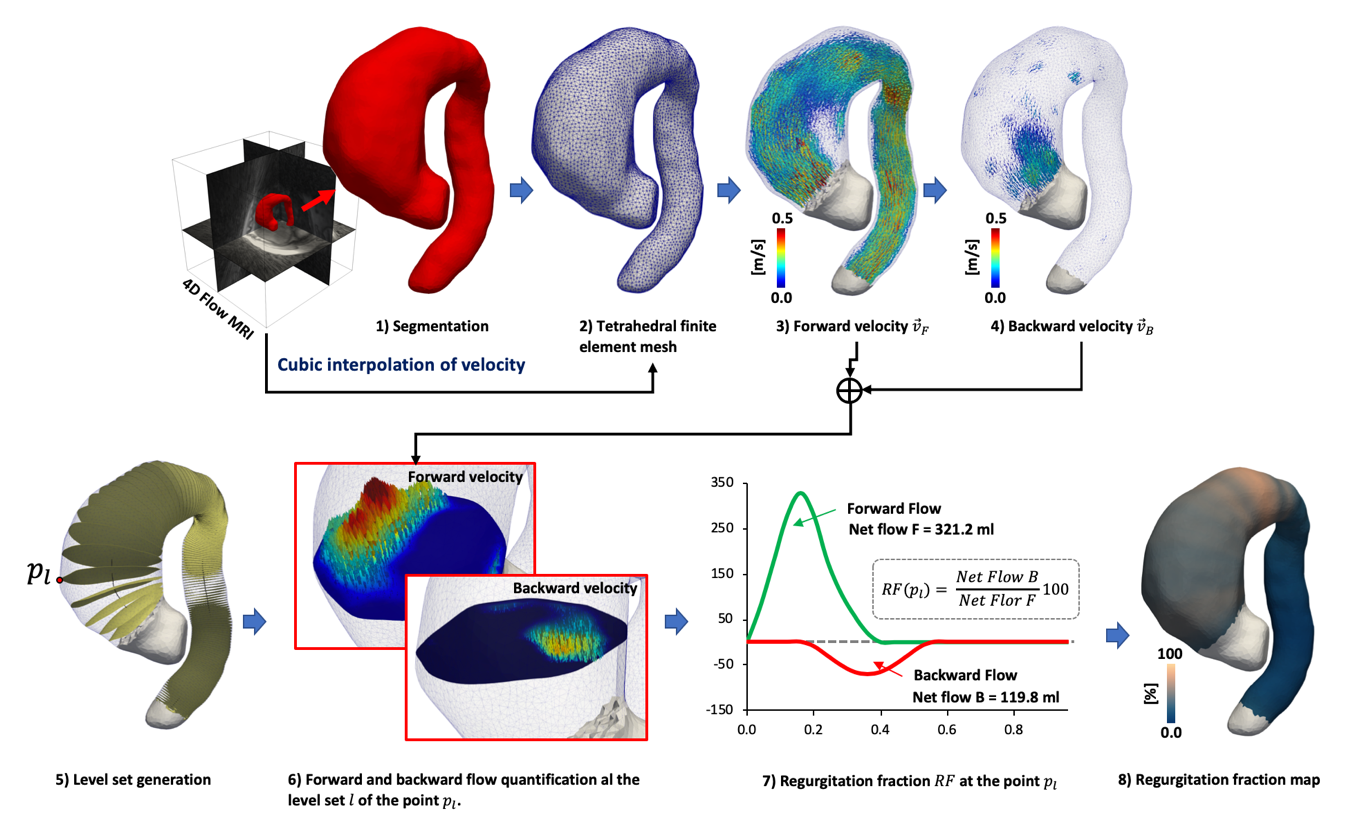


**Supplementary Figure 5.** Regurgitation fraction, measurement over each the level set generated by the Laplacian approach in each node of the surface mesh, was calculated using the forward and backward velocity vector field. Step 1: Segmentation of the vessel of interest from the 4D flow MRI data. Step 2: Generation of the tetrahedral finite element mesh. Step 3: Projection of the velocity in the forward direction using the axial unit vectors (see Figure 3). Step 4: Projection of the velocity in the backward direction using the backward unit vectors (see Figure 3). Step 5: Level set generated by the Laplacian solution. Step 6: Forward and Backward velocity for each level set. Step 7: Regurgitation fraction, calculate at the ratio between the net flow backward divided by the net flow forward. Step 8: Three-dimensional map of regurgitation fraction.


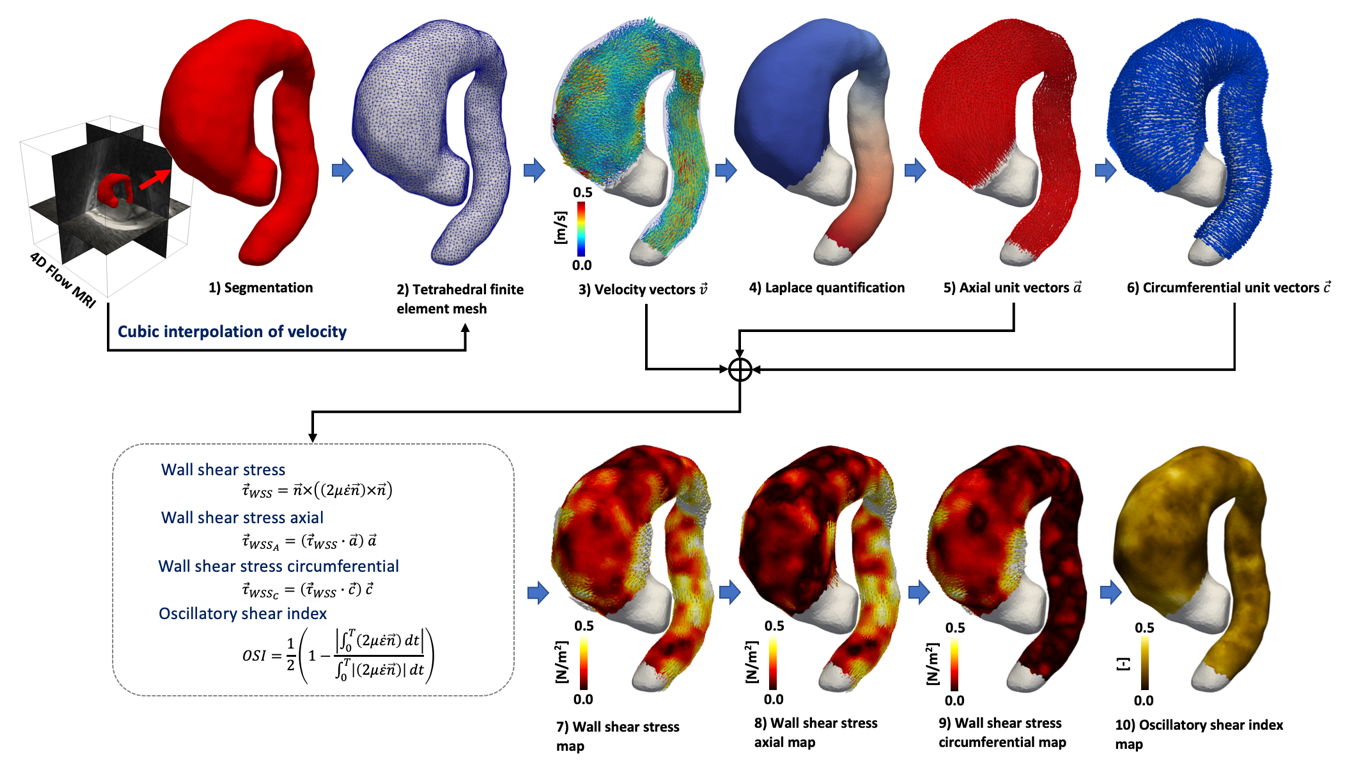


**Supplementary Figure 6.** Wall shear stress (WSS), axial WSS, circumferential WSS and oscillatory shear index, calculated using our finite element method and the Laplacian approach. Step 1: Segmentation of the vessel of interest from the 4D flow MRI data. Step 2: Generation of the tetrahedral finite element mesh. Step 3: Interpolation of the velocity to the tetrahedral finite element mesh. Step 4: Quantification of our Laplacian solution using the same boundary condition (Figure 1 Step 3). Step 5: Axial unit vector quantification, calculated as the normalized gradient of the Laplacian solution. Step 6: Circumferential unit vector quantification, calculated as the cross product between the axial and inward unit normal vector to each node in the surface. Step 7: Three-dimensional map of Wall Shear Stress vector. Step 8: Three-dimensional map of axial Wall Shear Stress vector. Step 9: Three-dimensional map of circumferential Wall Shear Stress vector. Step 10: Three-dimensional map of oscillatory shear index.


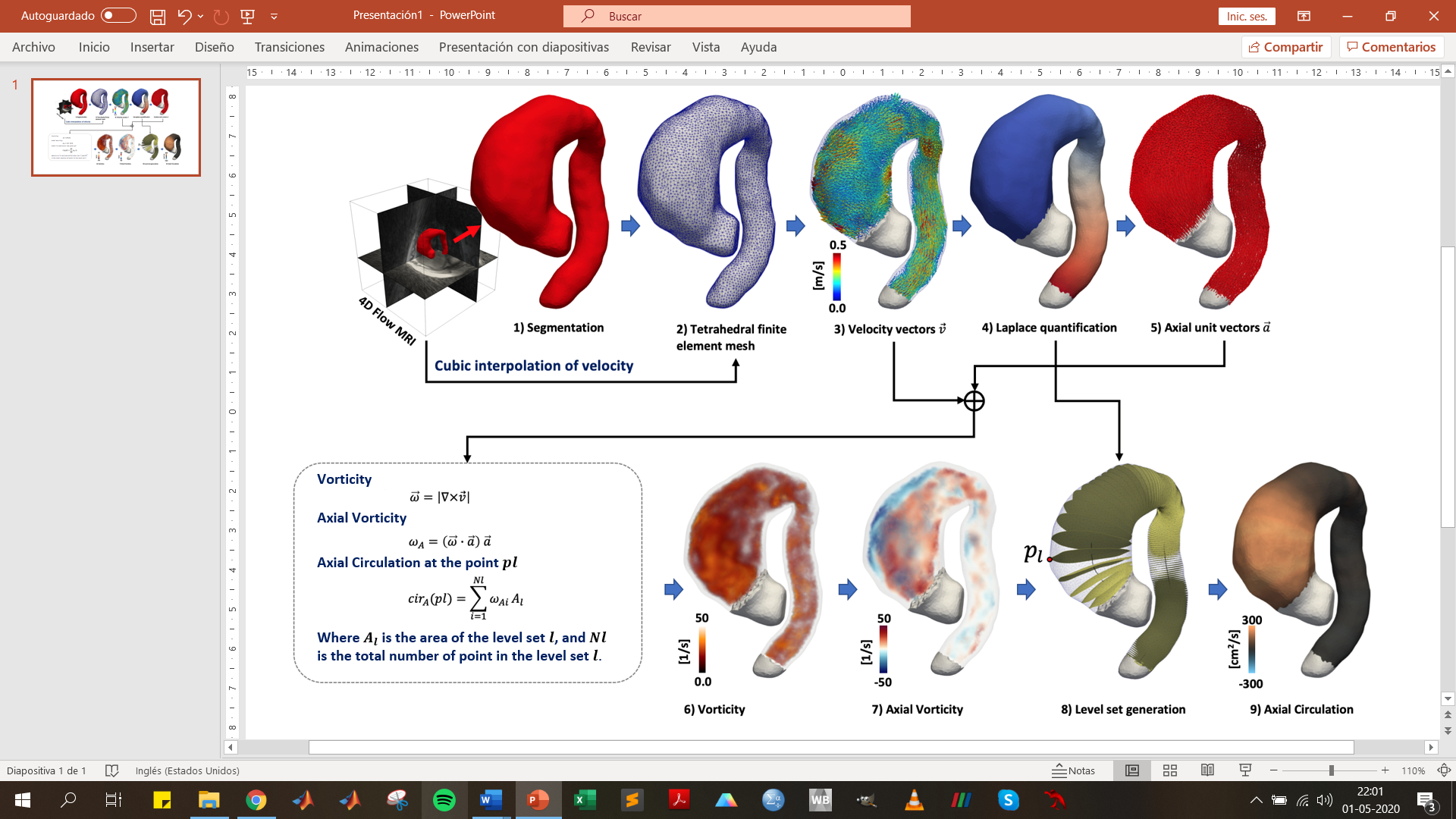


**Supplementary Figure 7.** The vorticity was calculated as the curl of velocity vectors field and its axial projection gave the axial vorticity, in three-dimensional domain. Finally, the axial circulation was calculated in each point of the surface of the vessel, as the area of the level set associated to each point multiplied by the sum of the axial vorticity for this particular level set. Step 1: Segmentation of the vessel of interest from the 4D flow MRI data. Step 2: Generation of the tetrahedral finite element mesh. Step 3: Interpolation of the velocity to the tetrahedral finite element mesh. Step 4: Quantification of our Laplacian solution using the same boundary condition (Figure 1 Step 3). Step 5: Axial unit vector quantification, calculated as the normalized gradient of the Laplacian solution. Step 6: Three-dimensional vorticity quantification. Step 7: Axial projection of the three-dimensional vorticity quantification (axial vorticity). Step 8: Level set generated by the Laplacian solution. Step 9: Three-dimensional axial circulation map.


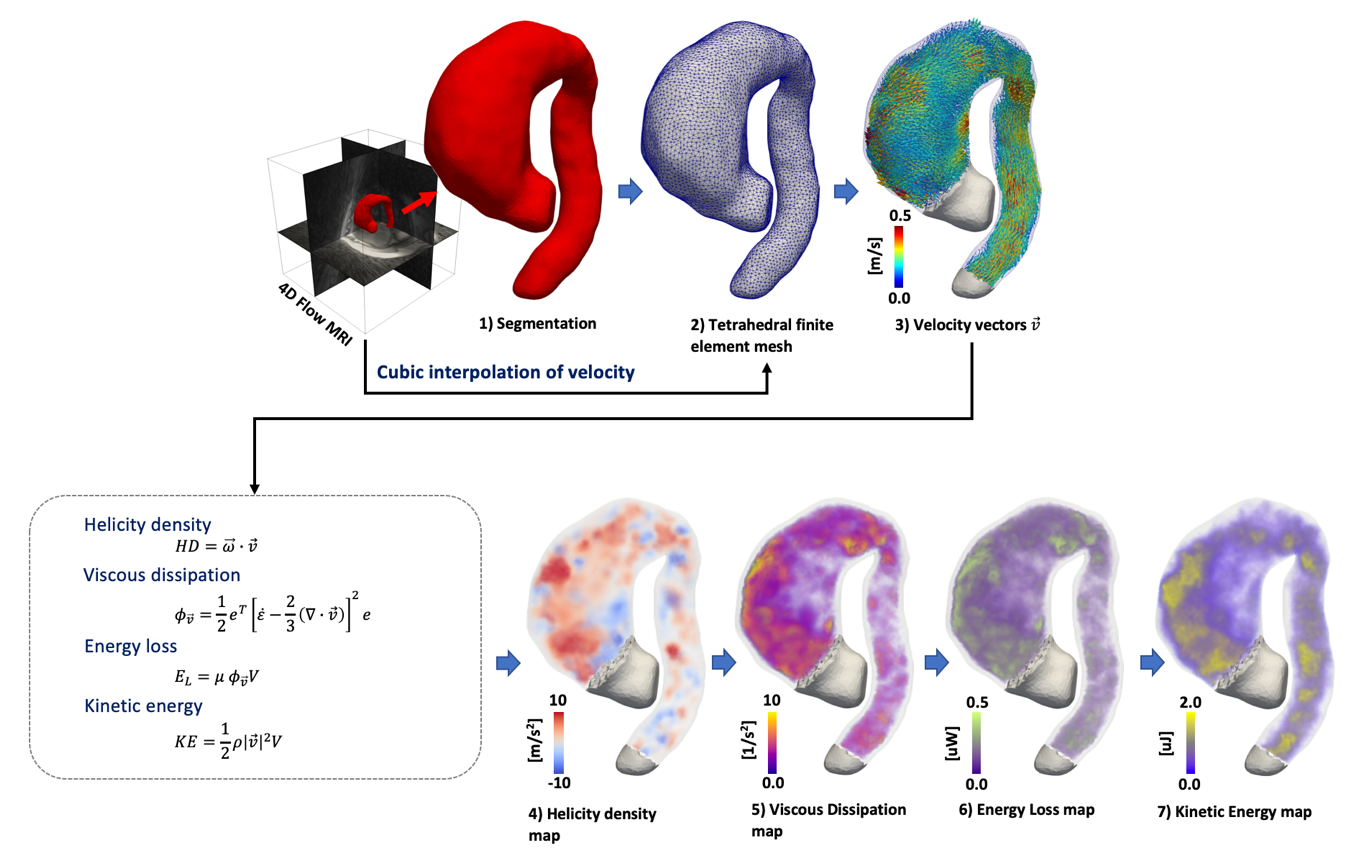


**Supplementary Figure 8.** The helicity density, viscous dissipation, energy loss and kinetic energy was calculated using our finite element approach. The two last depend on the Voronoi volume of each node of the tetrahedral finite element mesh. Step 1: Segmentation of the vessel of interest from the 4D flow MRI data. Step 2: Generation of the tetrahedral finite element mesh. Step 3: Interpolation of the velocity to the tetrahedral finite element mesh. Step 4: Three-dimensional helicity density quantification. Step 5: Three-dimensional viscous dissipation quantification. Step 6: Three-dimensional energy loss quantification. Step 7: Three-dimensional kinetic energy quantification.


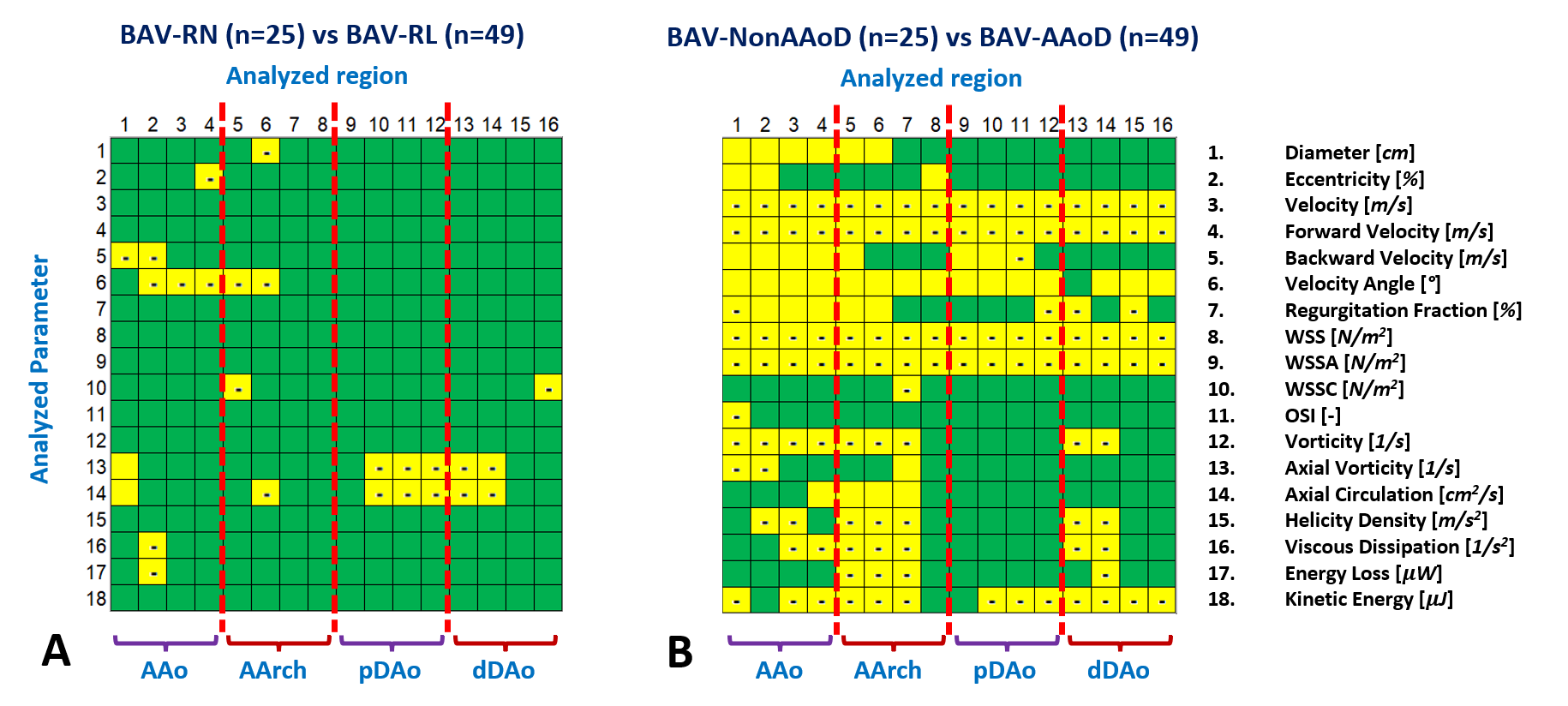


**Supplementary Figure 9**. A) Statistical differences between morphotypes groups; B) Statistical differences between BAV-NonAAoD and BAV-AAoD groups. Yellow marks highlight statistically significant differences between compared groups (p-value < 0.05). The minus sign in yellow boxes indicates the value was lower in BAV than in volunteers.


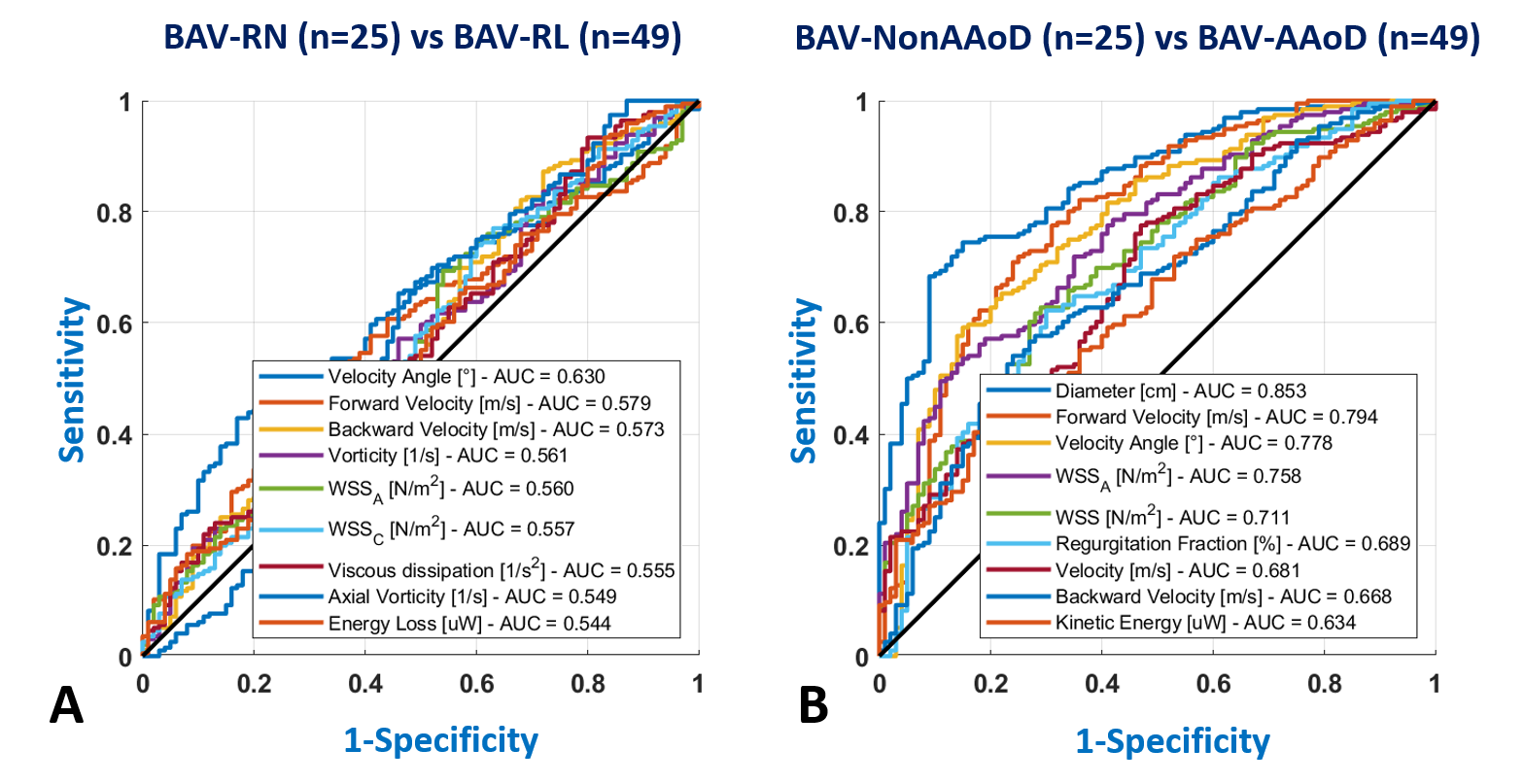


**Supplementary Figure 10**. ROC curves for the AAo; A) BAV-RN vs. BAV-RL; B) BAV-NonAAoD vs. BAV-AAoD. The parameters of the legend are the 9 parameters with bigger AUC (Area under the curve) values.


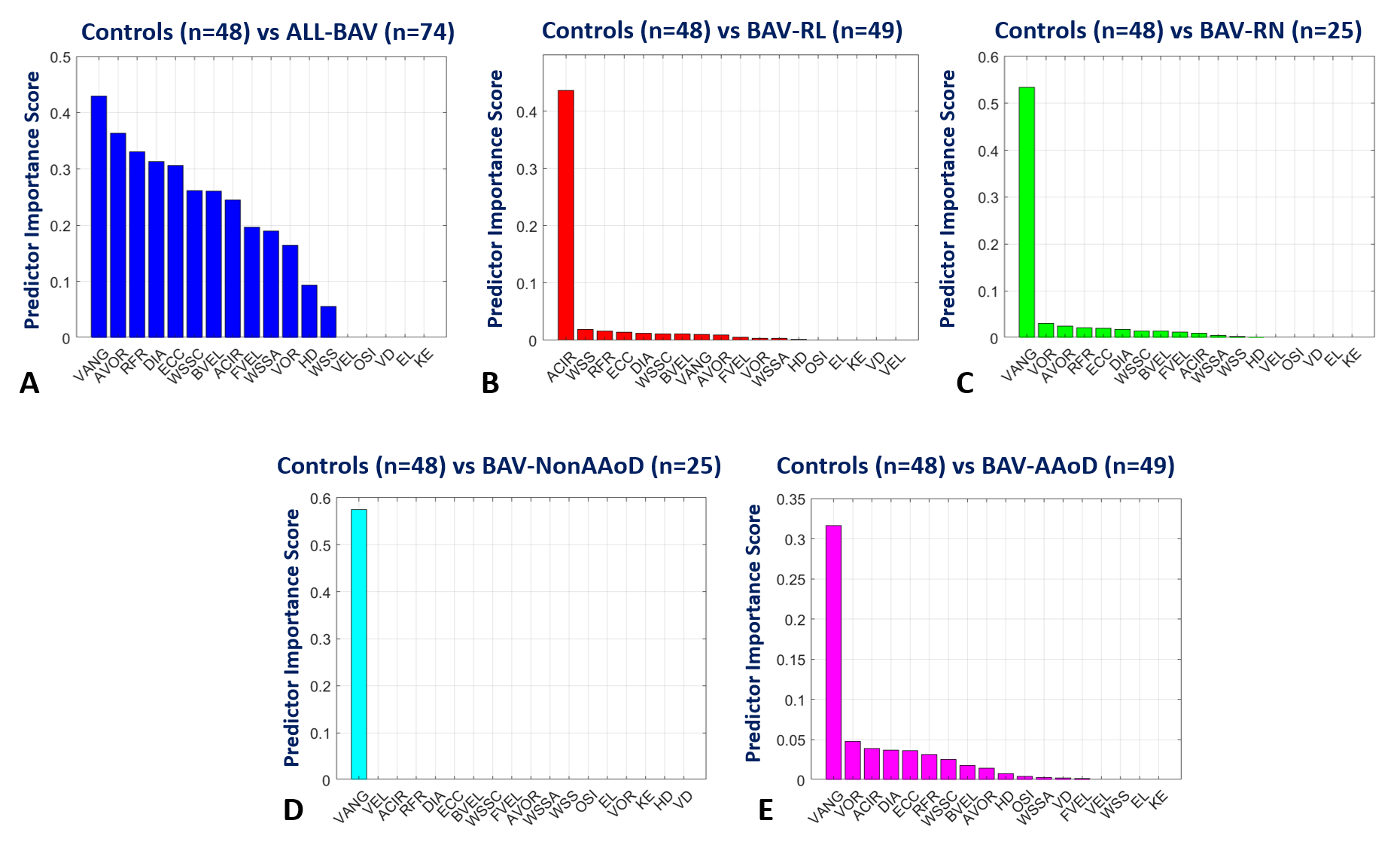


**Supplementary Figure 11**. Minimum redundancy maximum relevance classification algorithm. We show the parameters with bigger importance predictor score, to identify relevant hemodynamics parameters between controls and patients groups (ALL-BAV, BAV-RL, BAV-RN, BAV-NonAAoD, BAV-AAoD).


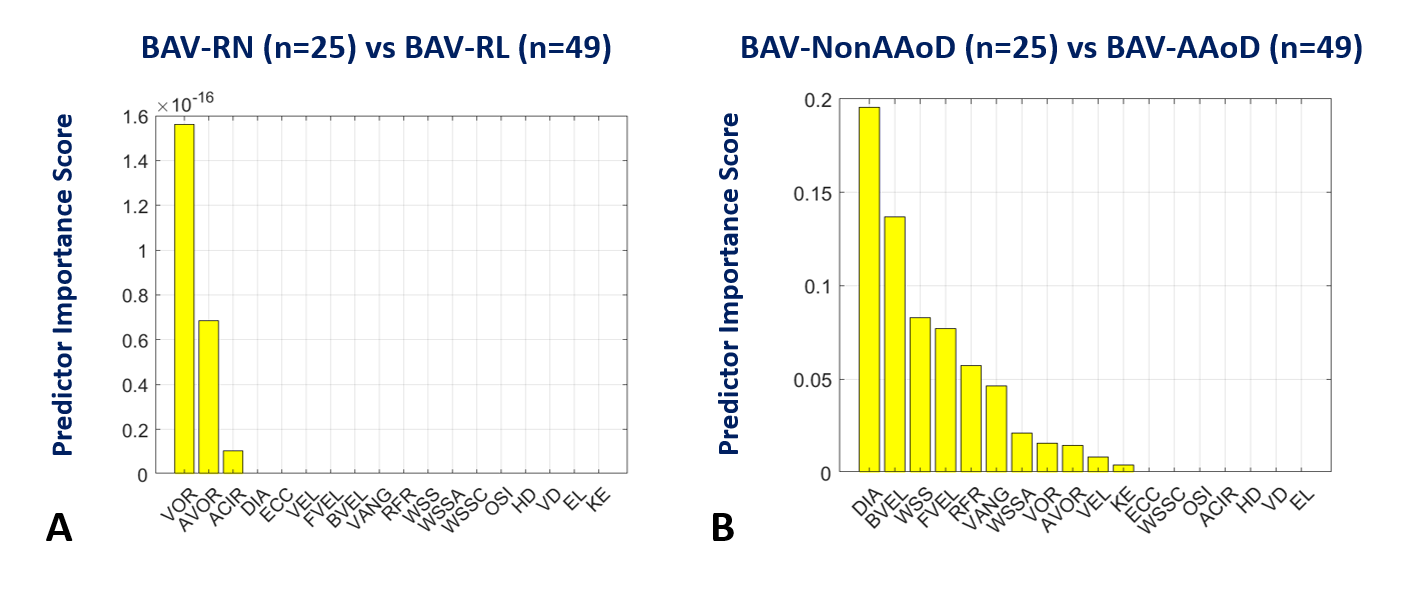


**Supplementary Figure 12**. Minimum redundancy maximum relevance classification algorithm in the AAo for morphotypes and phenotypes. We show the parameters with bigger importance predictor score, to identify relevant hemodynamics parameters between BAV-RN and BAV-RL groups (A) and between BAV-NonAAoD and BAV-AAoD groups (B).

| **Supplementary Table 1:** Mean and (standard deviation) for each parameter in the AArch section (region 5 to 8). The mark (*) show if the parameter is lower in the BAV than Volunteer groups. | | | | | | |
| --- | --- | --- | --- | --- | --- | --- |
| **Parameter** | **Volunteer** | **BAV**  **ALL** | **BAV**  **RN** | **BAV**  **RL** | **BAV**  **AAoD** | **BAV**  **NonAAoD** |
|  |  |  |  |  |  |  |
| Diameter [cm] | 2.41(0.30) | 2.63(0.66) | 2.57(0.64) | 2.75(0.67) | 2.71(0.69) | 2.47(0.56) |
| Eccentricity [%] | 16.16(7.71) | 21.74(9.94) | 21.30(10.31) | 22.59(9.16) | 22.55(9.14) | 20.14(11.22) |
| Velocity [m/s] | 0.38(0.12) | **0.35(0.12)*** | **0.35(0.11) *** | **0.34(0.12) *** | **0.32(0.10) *** | 0.40(0.12) |
| Forward Velocity [m/s] | 0.36(0.12) | **0.31(0.12) *** | **0.31(0.12) *** | **0.29(0.12) *** | **0.27(0.10) *** | 0.37(0.13) |
| Backward Velocity [m/s] | 0.00(0.00) | 0.00(0.01) | 0.00(0.01) | 0.00(0.00) | 0.00(0.01) | 0.00(0.01) |
| Velocity Angle [°] | 15.06(6.19) | 22.00(12.32) | 20.59(12.12) | 24.77(12.30) | 23.61(11.07) | 18.84(13.99) |
| Regurgitation Fraction [%] | 10.18(7.76) | 23.95(16.77) | 23.76(18.11) | 24.32(13.84) | 25.73(15.24) | 20.44(19.01) |
| WSS [N/m^2^] | 0.69(0.24) | **0.62(0.28) *** | **0.63(0.26) *** | **0.61(0.30) *** | **0.56(0.23) *** | 0.75(0.31) |
| WSSA [N/m^2^] | 0.66(0.23) | **0.57(0.28) *** | **0.59(0.27) *** | **0.54(0.31) *** | **0.50(0.23) *** | 0.70(0.32) |
| WSSC [N/m^2^] | 0.14(0.06) | 0.16(0.10) | 0.15(0.07) | 0.19(0.14) | 0.16(0.10) | 0.17(0.11) |
| OSI [-] | 0.14(0.06) | 0.18(0.06) | 0.18(0.06) | 0.17(0.05) | 0.18(0.05) | 0.17(0.06) |
| Vorticity [1/s] | 55.88(17.88) | **50.27(16.14) *** | **49.58(15.36) *** | **51.62(17.58) *** | **47.86(15.68) *** | **55.00(16.07) *** |
| Axial Vorticity [1/s] | 10.49(11.24) | **9.69(12.58) *** | **8.04(10.22) *** | 12.91(15.81) | **9.98(11.11) *** | **9.10(15.10) *** |
| Axial Circulation [cm^2^/s] | 46.14(47.56) | 63.63(105.59) | 48.22(77.05) | 93.83(141.91) | 69.73(101.48) | 51.67(112.78) |
| Helicity Density [m/s^2^] | 7.77(4.82) | **6.06(3.86) *** | **5.76(3.18) *** | **6.65(4.90) *** | **5.40(3.06) *** | **7.34(4.85) *** |
| Viscous Dissipation [1^3^/s^2^] | 5.03(2.86) | **4.56(2.68) *** | **4.55(2.60) *** | **4.59(2.84) *** | **4.06(2.42) *** | 5.56(2.89) |
| Energy Loss [uW] | 0.32(0.19) | **0.28(0.17) *** | **0.28(0.16) *** | **0.28(0.18) *** | **0.25(0.16) *** | 0.33(0.18) |
| Kinetic Energy [uJ] | 1.67(1.01) | **1.45(0.92) *** | **1.48(0.91) *** | **1.38(0.94) *** | **1.23(0.77) *** | 1.86(1.05) |
|  |  |  |  |  |  |  |

| **Supplementary Table 2:** Mean and (standard deviation) for each parameter in the pDAo section (region 9 to 12). The mark (*) show if the parameter is lower in the BAV than Volunteer groups. | | | | | | |
| --- | --- | --- | --- | --- | --- | --- |
| **Parameter** | **Volunteer** | **BAV**  **ALL** | **BAV**  **RN** | **BAV**  **RL** | **BAV**  **AAoD** | **BAV**  **NonAAoD** |
|  |  |  |  |  |  |  |
| Diameter [cm] | 2.28(0.30) | 2.43(0.44) | 2.42(0.44) | 2.44(0.44) | 2.47(0.43) | 2.34(0.45) |
| Eccentricity [%] | 14.34(6.82) | 18.36(8.41) | 19.04(7.87) | 17.03(9.28) | 18.94(8.26) | 17.25(8.62) |
| Velocity [m/s] | 0.42(0.11) | **0.37(0.12)*** | **0.36(0.11) *** | **0.38(0.13) *** | **0.34(0.10) *** | **0.42(0.13) *** |
| Forward Velocity [m/s] | 0.41(0.11) | **0.35(0.12) *** | **0.35(0.12) *** | **0.36(0.13) *** | **0.33(0.10) *** | **0.41(0.14) *** |
| Backward Velocity [m/s] | 0.00(0.00) | 0.00(0.00) | 0.00(0.00) | 0.00(0.00) | 0.00(0.00) | 0.00(0.00) |
| Velocity Angle [°] | 8.39(4.89) | 12.65(7.67) | 12.61(7.86) | 12.74(7.33) | 13.68(6.99) | 10.63(8.54) |
| Regurgitation Fraction [%] | 5.26(5.19) | 11.15(10.90) | 11.62(11.86) | 10.23(8.72) | 10.19(8.69) | 13.03(14.14) |
| WSS [N/m^2^] | 0.75(0.23) | **0.66(0.28) *** | **0.65(0.25) *** | **0.67(0.34) *** | **0.59(0.22) *** | 0.79(0.35) |
| WSSA [N/m^2^] | 0.74(0.23) | **0.64(0.29) *** | **0.63(0.25) *** | **0.66(0.34) *** | **0.57(0.21) *** | 0.77(0.36) |
| WSSC [N/m^2^] | 0.09(0.03) | 0.10(0.04) | 0.10(0.04) | 0.10(0.04) | 0.10(0.04) | 0.09(0.04) |
| OSI [-] | 0.10(0.05) | 0.13(0.05) | 0.13(0.05) | 0.13(0.04) | 0.13(0.04) | 0.14(0.05) |
| Vorticity [1/s] | 55.18(18.78) | **49.25(16.78) *** | **48.76(16.95) *** | **50.22(16.49) *** | **48.32(17.42) *** | **51.08(15.36) *** |
| Axial Vorticity [1/s] | -0.90(5.77) | **-3.22(8.34) *** | **-4.22(7.54) *** | **-1.26(9.46) *** | **-3.30(8.24) *** | **-3.06(8.59) *** |
| Axial Circulation [cm^2^/s] | -5.34(28.12) | **-19.14(40.05) *** | **-23.60(36.48) *** | **-10.39(45.19) *** | **-20.65(41.79) *** | **-16.18(36.41) *** |
| Helicity Density [m/s^2^] | 5.32(2.65) | **4.65(3.01) *** | **4.41(2.41) *** | **5.14(3.91) *** | **4.50(3.16) *** | **4.96(2.69) *** |
| Viscous Dissipation [1^3^/s^2^] | 5.05(3.16) | **3.80(2.03) *** | **3.72(2.01) *** | **3.95(2.06) *** | **3.65(2.05) *** | **4.09(1.97) *** |
| Energy Loss [uW] | 0.32(0.21) | **0.23(0.13) *** | **0.23(0.13) *** | **0.24(0.13) *** | **0.23(0.13) *** | **0.24(0.12) *** |
| Kinetic Energy [uJ] | 2.05(1.08) | **1.65(0.99) *** | **1.61(0.89) *** | **1.74(1.16) *** | **1.45(0.84) *** | 2.06(1.14) |
|  |  |  |  |  |  |  |

| **Supplementary Table 3:** Mean and (standard deviation) for each parameter in the dDAo section (region 13 to 16). The mark (*) show if the parameter is lower in the BAV than Volunteer groups. | | | | | | |
| --- | --- | --- | --- | --- | --- | --- |
| **Parameter** | **Volunteer** | **BAV**  **ALL** | **BAV**  **RN** | **BAV**  **RL** | **BAV**  **AAoD** | **BAV**  **NonAAoD** |
|  |  |  |  |  |  |  |
| Diameter [cm] | 2.08(0.30) | 2.29(0.41) | 2.30(0.42) | 2.28(0.37) | 2.31(0.39) | 2.26(0.44) |
| Eccentricity [%] | 14.18(6.72) | 14.91(5.85) | 14.43(5.17) | 15.86(6.92) | 15.07(5.77) | 14.60(6.02) |
| Velocity [m/s] | 0.40(0.12) | **0.36(0.11)*** | **0.35(0.11) *** | **0.37(0.12) *** | **0.33(0.09) *** | 0.42(0.13) |
| Forward Velocity [m/s] | 0.40(0.12) | **0.35(0.12) *** | **0.35(0.11) *** | **0.36(0.12) *** | **0.32(0.10) *** | 0.41(0.13) |
| Backward Velocity [m/s] | 0.00(0.00) | **0.00(0.00) *** | **0.00(0.00) *** | **0.00(0.00) *** | **0.00(0.00) *** | **0.00(0.00) *** |
| Velocity Angle [°] | 8.57(5.17) | 10.00(5.03) | 9.82(4.68) | 10.36(5.67) | 10.43(4.10) | 9.17(6.43) |
| Regurgitation Fraction [%] | 5.35(5.87) | 6.98(5.64) | 6.67(5.31) | 7.60(6.21) | 5.64(3.35) | 9.62(7.87) |
| WSS [N/m^2^] | 0.75(0.26) | **0.65(0.27) *** | **0.63(0.24) *** | **0.68(0.32) *** | **0.58(0.21) *** | 0.78(0.32) |
| WSSA [N/m^2^] | 0.74(0.26) | **0.64(0.27) *** | **0.62(0.24) *** | **0.67(0.33) *** | **0.57(0.22) *** | 0.77(0.32) |
| WSSC [N/m^2^] | 0.07(0.02) | 0.08(0.02) | 0.07(0.02) | 0.08(0.02) | 0.08(0.02) | 0.08(0.02) |
| OSI [-] | 0.10(0.06) | 0.12(0.05) | 0.12(0.05) | 0.12(0.05) | 0.12(0.04) | 0.13(0.05) |
| Vorticity [1/s] | 56.05(19.52) | **45.61(13.11) *** | **44.68(13.64) *** | **47.42(11.85) *** | **43.06(10.96) *** | **50.60(15.40) *** |
| Axial Vorticity [1/s] | -0.94(3.72) | **-2.86(5.45) *** | **-3.58(4.66) *** | **-1.44(6.53) *** | **-2.87(4.90) *** | **-2.83(6.41) *** |
| Axial Circulation [cm^2^/s] | -3.42(12.86) | **-10.64(17.53) *** | **-13.46(15.31) *** | **-5.12(20.19) *** | **-10.44(16.25) *** | **-11.04(19.87) *** |
| Helicity Density [m/s^2^] | 4.34(2.29) | **3.67(1.83) *** | **3.52(1.78) *** | **3.96(1.90) *** | **3.29(1.48) *** | 4.40(2.20) |
| Viscous Dissipation [1^3^/s^2^] | 5.75(3.74) | **3.49(1.88) *** | **3.36(1.91) *** | **3.74(1.82) *** | **3.12(1.61) *** | **4.22(2.16) *** |
| Energy Loss [uW] | 0.36(0.25) | **0.21(0.12) *** | **0.21(0.12) *** | **0.23(0.12) *** | **0.19(0.11) *** | **0.25(0.14) *** |
| Kinetic Energy [uJ] | 2.01(1.20) | **1.59(0.93) *** | **1.54(0.87) *** | **1.69(1.04) *** | **1.36(0.76) *** | 2.06(1.05) |
|  |  |  |  |  |  |  |

| **Supplementary Table 4:** Area under ROC curve for each parameter analyzed in this study. This analysis was performed using the regions 1 to 4 (AAo). | | | | | | | |
| --- | --- | --- | --- | --- | --- | --- | --- |
| **Parameter** | **Volunteer**  **vs**  **BAV-ALL** | **Volunteer**  **vs**  **BAV-RN** | **Volunteer**  **vs**  **BAV-RL** | **Volunteer**  **vs**  **BAV-AAoD** | **Volunteer**  **vs**  **BAV-NonAAoD** | **BAV-RN**  **vs**  **BAV-RL** | **BAV-NonAAoD**  **vs**  **BAV-AAoD** |
|  |  |  |  |  |  |  |  |
| Diameter [cm] | **0.926*** | **0.942*** | **0.919*** | **0.978*** | **0.826*** | 0.535 | **0.853*** |
| Eccentricity [%] | **0.906*** | **0.939*** | **0.890*** | **0.924*** | **0.871*** | 0.537 | 0.605 |
| Velocity [m/s] | 0.549 | 0.552 | 0.547 | 0.602 | 0.556 | 0.492 | 0.681 |
| Forward Velocity [m/s] | 0.786 | **0.832*** | 0.762 | **0.866*** | 0.629 | 0.579 | 0.794 |
| Backward Velocity [m/s] | **0.919*** | **0.940*** | **0.909*** | **0.949*** | **0.860*** | 0.573 | 0.668 |
| Velocity Angle [°] | **0.961*** | **0.987*** | **0.948*** | **0.992*** | **0.901*** | 0.630 | 0.778 |
| Regurgitation Fraction [%] | **0.940*** | **0.966*** | **0.927*** | **0.967*** | **0.887*** | 0.464 | 0.689 |
| WSS [N/m^2^] | 0.611 | 0.619 | 0.607 | 0.678 | 0.521 | 0.505 | 0.711 |
| WSS_A_ [N/m^2^] | 0.746 | 0.783 | 0.726 | **0.818*** | 0.604 | 0.560 | 0.758 |
| WSS_C_ [N/m^2^] | **0.898*** | **0.916*** | **0.889*** | **0.893*** | **0.908*** | 0.557 | 0.526 |
| OSI [-] | 0.534 | 0.524 | 0.538 | 0.492 | 0.584 | 0.510 | 0.605 |
| Vorticity [1/s] | 0.620 | 0.663 | 0.598 | 0.567 | 0.725 | 0.561 | 0.633 |
| Axial Vorticity [1/s] | **0.855*** | 0.798 | **0.883*** | **0.859*** | **0.846*** | 0.549 | 0.596 |
| Axial Circulation [cm^2^/s] | **0.935*** | **0.887*** | **0.960*** | **0.955*** | **0.897*** | 0.481 | 0.575 |
| Helicity Density [m/s^2^] | 0.596 | 0.625 | 0.582 | 0.556 | 0.675 | 0.539 | 0.593 |
| Viscous Dissipation [1/s^2^] | 0.592 | 0.627 | 0.575 | 0.559 | 0.658 | 0.555 | 0.582 |
| Energy Loss [uW] | 0.582 | 0.612 | 0.566 | 0.558 | 0.627 | 0.544 | 0.561 |
| Kinetic Energy [uJ] | 0.507 | 0.511 | 0.505 | 0.548 | 0.575 | 0.512 | 0.634 |
|  |  |  |  |  |  |  |  |
| * Area under ROC curve bigger than 0.8 | | | | | | | |

| **Supplementary Table 5:** Area under ROC curve for each parameter analyzed in this study. This analysis was performed using the regions 5 to 8 (AArch). | | | | | | | |
| --- | --- | --- | --- | --- | --- | --- | --- |
| **Parameter** | **Volunteer**  **vs**  **BAV-ALL** | **Volunteer**  **vs**  **BAV-RN** | **Volunteer**  **vs**  **BAV-RL** | **Volunteer**  **vs**  **BAV-AAoD** | **Volunteer**  **vs**  **BAV-NonAAoD** | **BAV-RN**  **vs**  **BAV-RL** | **BAV-NonAAoD**  **vs**  **BAV-AAoD** |
|  |  |  |  |  |  |  |  |
| Diameter [cm] | 0.604 | 0.691 | 0.560 | 0.642 | 0.530 | 0.596 | 0.605 |
| Eccentricity [%] | 0.679 | 0.715 | 0.661 | 0.718 | 0.603 | 0.561 | 0.615 |
| Velocity [m/s] | 0.588 | 0.602 | 0.581 | 0.659 | 0.553 | 0.523 | 0.707 |
| Forward Velocity [m/s] | 0.633 | 0.669 | 0.614 | 0.711 | 0.521 | 0.559 | 0.715 |
| Backward Velocity [m/s] | 0.652 | 0.685 | 0.635 | 0.684 | 0.590 | 0.458 | 0.594 |
| Velocity Angle [°] | 0.680 | 0.755 | 0.642 | 0.750 | 0.543 | 0.613 | 0.676 |
| Regurgitation Fraction [%] | 0.790 | **0.820*** | 0.774 | **0.833*** | 0.705 | 0.539 | 0.634 |
| WSS [N/m^2^] | 0.598 | 0.627 | 0.583 | 0.665 | 0.534 | 0.547 | 0.688 |
| WSS_A_ [N/m^2^] | 0.628 | 0.681 | 0.602 | 0.697 | 0.505 | 0.585 | 0.686 |
| WSS_C_ [N/m^2^] | 0.551 | 0.601 | 0.525 | 0.542 | 0.568 | 0.583 | 0.529 |
| OSI [-] | 0.705 | 0.690 | 0.713 | 0.720 | 0.677 | 0.560 | 0.510 |
| Vorticity [1/s] | 0.605 | 0.589 | 0.613 | 0.645 | 0.526 | 0.532 | 0.647 |
| Axial Vorticity [1/s] | 0.523 | 0.528 | 0.549 | 0.503 | 0.561 | 0.574 | 0.561 |
| Axial Circulation [cm^2^/s] | 0.503 | 0.567 | 0.471 | 0.540 | 0.432 | 0.587 | 0.598 |
| Helicity Density [m/s^2^] | 0.613 | 0.583 | 0.628 | 0.656 | 0.528 | 0.548 | 0.642 |
| Viscous Dissipation [1/s^2^] | 0.558 | 0.564 | 0.555 | 0.615 | 0.552 | 0.501 | 0.688 |
| Energy Loss [uW] | 0.569 | 0.578 | 0.565 | 0.616 | 0.522 | 0.503 | 0.660 |
| Kinetic Energy [uJ] | 0.563 | 0.586 | 0.551 | 0.627 | 0.563 | 0.539 | 0.688 |
|  |  |  |  |  |  |  |  |
| * Area under ROC curve bigger than 0.8 | | | | | | | |

| **Supplementary Table 6:** Area under ROC curve for each parameter analyzed in this study. This analysis was performed using the regions 9 to 12 (pDAo). | | | | | | | |
| --- | --- | --- | --- | --- | --- | --- | --- |
| **Parameter** | **Volunteer**  **vs**  **BAV-ALL** | **Volunteer**  **vs**  **BAV-RN** | **Volunteer**  **vs**  **BAV-RL** | **Volunteer**  **vs**  **BAV-AAoD** | **Volunteer**  **vs**  **BAV-NonAAoD** | **BAV-RN**  **vs**  **BAV-RL** | **BAV-NonAAoD**  **vs**  **BAV-AAoD** |
|  |  |  |  |  |  |  |  |
| Diameter [cm] | 0.618 | 0.640 | 0.607 | 0.651 | 0.552 | 0.510 | 0.593 |
| Eccentricity [%] | 0.646 | 0.572 | 0.684 | 0.670 | 0.599 | 0.593 | 0.568 |
| Velocity [m/s] | 0.635 | 0.612 | 0.647 | 0.703 | 0.501 | 0.534 | 0.684 |
| Forward Velocity [m/s] | 0.647 | 0.628 | 0.657 | 0.722 | 0.501 | 0.534 | 0.697 |
| Backward Velocity [m/s] | 0.624 | 0.636 | 0.618 | 0.678 | 0.518 | 0.512 | 0.658 |
| Velocity Angle [°] | 0.711 | 0.732 | 0.701 | 0.789 | 0.559 | 0.514 | 0.705 |
| Regurgitation Fraction [%] | 0.728 | 0.712 | 0.737 | 0.709 | 0.767 | 0.536 | 0.571 |
| WSS [N/m^2^] | 0.644 | 0.648 | 0.642 | 0.718 | 0.501 | 0.497 | 0.675 |
| WSS_A_ [N/m^2^] | 0.652 | 0.657 | 0.650 | 0.729 | 0.499 | 0.499 | 0.684 |
| WSS_C_ [N/m^2^] | 0.568 | 0.599 | 0.551 | 0.582 | 0.539 | 0.544 | 0.542 |
| OSI [-] | 0.726 | 0.722 | 0.728 | 0.721 | 0.735 | 0.536 | 0.570 |
| Vorticity [1/s] | 0.598 | 0.584 | 0.604 | 0.616 | 0.562 | 0.534 | 0.567 |
| Axial Vorticity [1/s] | 0.578 | 0.459 | 0.639 | 0.568 | 0.598 | 0.647 | 0.478 |
| Axial Circulation [cm^2^/s] | 0.602 | 0.473 | 0.668 | 0.594 | 0.618 | 0.650 | 0.492 |
| Helicity Density [m/s^2^] | 0.602 | 0.571 | 0.618 | 0.625 | 0.558 | 0.549 | 0.575 |
| Viscous Dissipation [1/s^2^] | 0.617 | 0.600 | 0.626 | 0.639 | 0.575 | 0.535 | 0.575 |
| Energy Loss [uW] | 0.621 | 0.607 | 0.627 | 0.632 | 0.599 | 0.531 | 0.547 |
| Kinetic Energy [uJ] | 0.607 | 0.590 | 0.616 | 0.665 | 0.506 | 0.523 | 0.660 |
|  |  |  |  |  |  |  |  |
| * Area under ROC curve bigger than 0.8 | | | | | | | |

| **Supplementary Table 7:** Area under ROC curve for each parameter analyzed in this study. This analysis was performed using the regions 13 to 16 (dDAo). | | | | | | | |
| --- | --- | --- | --- | --- | --- | --- | --- |
| **Parameter** | **Volunteer**  **vs**  **BAV-ALL** | **Volunteer**  **vs**  **BAV-RN** | **Volunteer**  **vs**  **BAV-RL** | **Volunteer**  **vs**  **BAV-AAoD** | **Volunteer**  **vs**  **BAV-NonAAoD** | **BAV-RN**  **vs**  **BAV-RL** | **BAV-NonAAoD**  **vs**  **BAV-AAoD** |
|  |  |  |  |  |  |  |  |
| Diameter [cm] | 0.678 | 0.692 | 0.671 | 0.691 | 0.652 | 0.514 | 0.537 |
| Eccentricity [%] | 0.556 | 0.589 | 0.539 | 0.564 | 0.539 | 0.570 | 0.527 |
| Velocity [m/s] | 0.602 | 0.568 | 0.620 | 0.676 | 0.542 | 0.559 | 0.711 |
| Forward Velocity [m/s] | 0.599 | 0.569 | 0.614 | 0.672 | 0.544 | 0.553 | 0.711 |
| Backward Velocity [m/s] | 0.498 | 0.479 | 0.507 | 0.472 | 0.549 | 0.542 | 0.410 |
| Velocity Angle [°] | 0.617 | 0.621 | 0.615 | 0.672 | 0.509 | 0.504 | 0.657 |
| Regurgitation Fraction [%] | 0.628 | 0.652 | 0.616 | 0.593 | 0.698 | 0.534 | 0.635 |
| WSS [N/m^2^] | 0.632 | 0.620 | 0.638 | 0.707 | 0.516 | 0.526 | 0.693 |
| WSS_A_ [N/m^2^] | 0.631 | 0.622 | 0.636 | 0.708 | 0.518 | 0.524 | 0.695 |
| WSS_C_ [N/m^2^] | 0.554 | 0.624 | 0.519 | 0.567 | 0.530 | 0.638 | 0.558 |
| OSI [-] | 0.658 | 0.677 | 0.647 | 0.642 | 0.688 | 0.506 | 0.577 |
| Vorticity [1/s] | 0.670 | 0.646 | 0.682 | 0.713 | 0.585 | 0.580 | 0.648 |
| Axial Vorticity [1/s] | 0.633 | 0.549 | 0.675 | 0.618 | 0.661 | 0.612 | 0.455 |
| Axial Circulation [cm^2^/s] | 0.650 | 0.554 | 0.699 | 0.632 | 0.686 | 0.629 | 0.550 |
| Helicity Density [m/s^2^] | 0.593 | 0.545 | 0.618 | 0.645 | 0.509 | 0.581 | 0.652 |
| Viscous Dissipation [1/s^2^] | 0.699 | 0.676 | 0.710 | 0.739 | 0.620 | 0.578 | 0.663 |
| Energy Loss [uW] | 0.698 | 0.678 | 0.708 | 0.729 | 0.636 | 0.562 | 0.629 |
| Kinetic Energy [uJ] | 0.600 | 0.571 | 0.616 | 0.669 | 0.534 | 0.544 | 0.704 |
|  |  |  |  |  |  |  |  |
| * Area under ROC curve bigger than 0.8 | | | | | | | |

| **Supplementary Table 8:** Predictor importance score: Rank features for classification using minimum redundancy maximum relevance (MRMR) algorithm, this parameter was analyzing using the regions 1 to 4 (AAo). | | | | | |
| --- | --- | --- | --- | --- | --- |
| **Parameter** | **Volunteer**  **vs**  **BAV-ALL** | **Volunteer**  **vs**  **BAV-RL** | **Volunteer**  **vs**  **BAV-RN** | **Volunteer**  **vs**  **BAV-AAoD** | **Volunteer**  **vs**  **BAV-NonAAoD** |
|  |  |  |  |  |  |
| Diameter [cm] | **0.313*** | **0.012*** | **0.018*** | 0.000 | **0.037*** |
| Eccentricity [%] | **0.307*** | **0.014*** | **0.021*** | 0.000 | **0.036*** |
| Velocity [m/s] | 0.000 | 0.000 | 0.000 | 0.000 | 0.000 |
| Forward Velocity [m/s] | **0.196*** | **0.005*** | **0.012*** | 0.000 | **0.001*** |
| Backward Velocity [m/s] | **0.261*** | **0.010*** | **0.014*** | 0.000 | **0.018*** |
| Velocity Angle [°] | **0.429*** | **0.010*** | **0.534*** | **0.575*** | **0.317*** |
| Regurgitation Fraction [%] | **0.331*** | **0.015*** | **0.022*** | 0.000 | **0.031*** |
| WSS [N/m^2^] | **0.056*** | **0.018*** | **0.003*** | 0.000 | 0.000 |
| WSS_A_ [N/m^2^] | **0.190*** | **0.003*** | **0.006*** | 0.000 | **0.003*** |
| WSS_C_ [N/m^2^] | **0.261*** | **0.011*** | **0.014*** | 0.000 | **0.025*** |
| OSI [-] | 0.000 | 0.000 | 0.000 | 0.000 | **0.004*** |
| Vorticity [1/s] | **0.164*** | **0.003*** | **0.031*** | 0.000 | **0.048*** |
| Axial Vorticity [1/s] | **0.364*** | **0.009*** | **0.026*** | 0.000 | **0.015*** |
| Axial Circulation [cm^2^/s] | **0.245*** | **0.437*** | **0.010*** | 0.000 | **0.039*** |
| Helicity Density [m/s^2^] | **0.093*** | **0.001*** | **0.001*** | 0.000 | **0.008*** |
| Viscous Dissipation [1/s^2^] | 0.000 | 0.000 | 0.000 | 0.000 | **0.002*** |
| Energy Loss [uW] | 0.000 | 0.000 | 0.000 | 0.000 | 0.000 |
| Kinetic Energy [uJ] | 0.000 | 0.000 | 0.000 | 0.000 | 0.000 |
|  |  |  |  |  |  |
| * Score ≠ 0.000 | | | | | |

| **Supplementary Table 9:** Predictor importance score: Rank features for classification using minimum redundancy maximum relevance (MRMR) algorithm, this parameter was analyzing using the regions 5 to 8 (AArch). | | | | | |
| --- | --- | --- | --- | --- | --- |
| **Parameter** | **Volunteer**  **vs**  **BAV-ALL** | **Volunteer**  **vs**  **BAV-RL** | **Volunteer**  **vs**  **BAV-RN** | **Volunteer**  **vs**  **BAV-AAoD** | **Volunteer**  **vs**  **BAV-NonAAoD** |
|  |  |  |  |  |  |
| Diameter [cm] | **0.007*** | 0.000 | **0.011*** | **0.007*** | 0.000 |
| Eccentricity [%] | **0.015*** | 0.000 | **0.020*** | **0.020*** | 0.000 |
| Velocity [m/s] | 0.000 | 0.000 | 0.000 | **0.001*** | 0.000 |
| Forward Velocity [m/s] | **0.001*** | 0.000 | **0.001*** | **0.002*** | 0.000 |
| Backward Velocity [m/s] | **0.001*** | 0.000 | **0.001*** | **0.002*** | **0.032*** |
| Velocity Angle [°] | **0.004*** | 0.000 | **0.005*** | **0.005*** | 0.000 |
| Regurgitation Fraction [%] | **0.121*** | **0.112*** | **0.143*** | **0.190*** | **0.060*** |
| WSS [N/m^2^] | **0.001*** | 0.000 | **0.000*** | **0.001*** | 0.000 |
| WSS_A_ [N/m^2^] | **0.001*** | 0.000 | **0.002*** | **0.001*** | 0.000 |
| WSS_C_ [N/m^2^] | 0.000 | 0.000 | 0.000 | 0.000 | 0.000 |
| OSI [-] | **0.003*** | 0.000 | **0.002*** | **0.003*** | **0.006*** |
| Vorticity [1/s] | **0.001*** | 0.000 | 0.000 | **0.001*** | 0.000 |
| Axial Vorticity [1/s] | 0.000 | 0.000 | 0.000 | 0.000 | 0.000 |
| Axial Circulation [cm^2^/s] | 0.000 | 0.000 | 0.000 | 0.000 | 0.000 |
| Helicity Density [m/s^2^] | 0.000 | 0.000 | 0.000 | **0.027*** | 0.000 |
| Viscous Dissipation [1/s^2^] | **0.021*** | 0.000 | **0.001*** | **0.003*** | 0.000 |
| Energy Loss [uW] | **0.001*** | 0.000 | **0.027*** | **0.001*** | 0.000 |
| Kinetic Energy [uJ] | 0.000 | 0.000 | 0.000 | 0.000 | 0.000 |
|  |  |  |  |  |  |
| * Score ≠ 0.000 | | | | | |

| **Supplementary Table 10:** Predictor importance score: Rank features for classification using minimum redundancy maximum relevance (MRMR) algorithm, this parameter was analyzing using the regions 9 to 12 (pDAo). | | | | | |
| --- | --- | --- | --- | --- | --- |
| **Parameter** | **Volunteer**  **vs**  **BAV-ALL** | **Volunteer**  **vs**  **BAV-RL** | **Volunteer**  **vs**  **BAV-RN** | **Volunteer**  **vs**  **BAV-AAoD** | **Volunteer**  **vs**  **BAV-NonAAoD** |
|  |  |  |  |  |  |
| Diameter [cm] | **0.005*** | 0.000 | **0.034*** | 0.000 | 0.000 |
| Eccentricity [%] | **0.012*** | 0.000 | 0.000 | 0.000 | 0.000 |
| Velocity [m/s] | 0.000 | 0.000 | 0.000 | 0.000 | 0.000 |
| Forward Velocity [m/s] | **0.002*** | 0.000 | **0.012*** | 0.000 | 0.000 |
| Backward Velocity [m/s] | **0.002*** | 0.000 | **0.032*** | 0.000 | **0.003*** |
| Velocity Angle [°] | **0.015*** | 0.000 | **0.062*** | **0.129*** | 0.000 |
| Regurgitation Fraction [%] | **0.007*** | 0.000 | **0.052*** | 0.000 | **0.113*** |
| WSS [N/m^2^] | **0.001*** | 0.000 | **0.008*** | 0.000 | 0.000 |
| WSS_A_ [N/m^2^] | **0.007*** | 0.000 | **0.033*** | 0.000 | 0.000 |
| WSS_C_ [N/m^2^] | 0.000 | 0.000 | 0.000 | 0.000 | 0.000 |
| OSI [-] | **0.085*** | **0.090*** | **0.030*** | 0.000 | **0.053*** |
| Vorticity [1/s] | **0.001*** | 0.000 | 0.000 | 0.000 | 0.000 |
| Axial Vorticity [1/s] | 0.000 | 0.000 | 0.000 | 0.000 | 0.000 |
| Axial Circulation [cm^2^/s] | 0.000 | 0.000 | 0.000 | 0.000 | 0.000 |
| Helicity Density [m/s^2^] | **0.003*** | 0.000 | 0.000 | **0.031*** | 0.000 |
| Viscous Dissipation [1/s^2^] | **0.001*** | 0.000 | 0.000 | 0.000 | 0.000 |
| Energy Loss [uW] | **0.019*** | 0.000 | 0.000 | 0.000 | 0.000 |
| Kinetic Energy [uJ] | 0.000 | 0.000 | 0.000 | 0.000 | 0.000 |
|  |  |  |  |  |  |
| * Score ≠ 0.000 | | | | | |

| **Supplementary Table 11:** Predictor importance score: Rank features for classification using minimum redundancy maximum relevance (MRMR) algorithm, this parameter was analyzing using the regions 13 to 16 (dDAo). | | | | | |
| --- | --- | --- | --- | --- | --- |
| **Parameter** | **Volunteer**  **vs**  **BAV-ALL** | **Volunteer**  **vs**  **BAV-RL** | **Volunteer**  **vs**  **BAV-RN** | **Volunteer**  **vs**  **BAV-AAoD** | **Volunteer**  **vs**  **BAV-NonAAoD** |
|  |  |  |  |  |  |
| Diameter [cm] | **0.018*** | 0.000 | **0.026*** | **0.010*** | **0.009*** |
| Eccentricity [%] | 0.000 | 0.000 | **0.022*** | 0.000 | 0.000 |
| Velocity [m/s] | 0.000 | 0.000 | 0.000 | **0.001*** | 0.000 |
| Forward Velocity [m/s] | 0.000 | 0.000 | 0.000 | **0.002*** | 0.000 |
| Backward Velocity [m/s] | **0.003*** | 0.000 | **0.004*** | **0.005*** | **0.001*** |
| Velocity Angle [°] | **0.020*** | 0.000 | 0.000 | **0.079*** | 0.000 |
| Regurgitation Fraction [%] | **0.004*** | **0.016*** | **0.002*** | **0.014*** | **0.043*** |
| WSS [N/m^2^] | **0.003*** | 0.000 | 0.000 | **0.002*** | 0.000 |
| WSS_A_ [N/m^2^] | **0.001*** | 0.000 | **0.001*** | **0.005*** | 0.000 |
| WSS_C_ [N/m^2^] | 0.000 | 0.000 | **0.040*** | 0.000 | 0.000 |
| OSI [-] | **0.037*** | 0.000 | **0.041*** | **0.003*** | **0.002*** |
| Vorticity [1/s] | **0.005*** | 0.000 | **0.002*** | **0.005*** | 0.000 |
| Axial Vorticity [1/s] | **0.003*** | 0.000 | 0.000 | 0.000 | **0.001*** |
| Axial Circulation [cm^2^/s] | **0.030*** | **0.050*** | 0.000 | **0.027*** | **0.038*** |
| Helicity Density [m/s^2^] | **0.001*** | 0.000 | 0.000 | **0.002*** | 0.000 |
| Viscous Dissipation [1/s^2^] | **0.005*** | **0.103*** | **0.001*** | **0.004*** | 0.000 |
| Energy Loss [uW] | **0.100*** | 0.000 | **0.050*** | **0.162*** | **0.024*** |
| Kinetic Energy [uJ] | 0.000 | 0.000 | 0.000 | **0.006*** | 0.000 |
|  |  |  |  |  |  |
| * Score ≠ 0.000 | | | | | |
